# Supplementary material for: A spin-crossover framework endowed with pore-adjustable behavior by slow structural dynamics
Source: Nat Commun. 2022 Jun 18;13:3510. doi: 10.1038/s41467-022-31274-8 (PMC9206640; doi:10.1038/s41467-022-31274-8)
Supplement: Supplementary file 1 — Supplementary Information [file 41467_2022_31274_MOESM1_ESM.pdf]

Supplementary Information

for

A spin-crossover framework endowed with pore-adjustable behavior by slow structural dynamics

Jin-Peng Xue, Yang Hu, Bo Zhao, Zhi-Kun Liu, Jing Xie, Zi-Shuo Yao* & Jun Tao*

Key Laboratory of Cluster Science of Ministry of Education, School of Chemistry and Chemical Engineering, Liangxiang Campus, Beijing Institute of Technology, Beijing 102488, People's Republic of China.

*email: zishuoyao@bit.edu.cn; taojun@bit.edu.cn

Table of Contents

Supplementary Methods S5

Synthesis of ligand (1*E*,2*E*)-3-phenyl-N-(4H-1,2,4-triazol-4-yl)prop-2-en-1-imine S5

Differential scanning calorimetry (DSC) S5

Thermogravimetric analyses (TGA)............ S5

Computational Details S5

Supplementary Figures S7

Supplementary Fig. 1 Thermogravimetric analysis of the pristine crystal (**1**·*x*MeOH·*y*H_2O) S7

Supplementary Fig. 2 Layer structure of **1**·9/2H_2O and **1**·4/3H_2O S7

Supplementary Fig. 3 The asymmetric unit of **1**·9/2H_2O (**a**) and **1**·4/3H_2O (**b**) S8

Supplementary Fig. 4 Pore structure and void space of **1**·9/2H_2O by Connolly surface S8

Supplementary Fig. 5 TGA of lcp phase **1**·9/2H_2O (**a**) and nqp phase **1**·4/3H_2O (**b**) S9

Supplementary Fig. 6 CO_2 adsorption of lcp **1**·9/2H_2O activated under mild conditions S9

Supplementary Fig. 7 Crystal structures and pore shapes of **1**·9/2H_2O and **1**·4/3H_2O S10

Supplementary Fig. 8 *In situ* PXRD patterns of **1** upon heating at 433 K for 48h under vacuum S10

Supplementary Fig. 9 Rietveld refinement and PXRD patterns of lcp and nqp phases at 250 K S11

Supplementary Fig. 10 The water ad/de-sorption measurements activated at different temperatures S12

Supplementary Fig. 11 PXRD patterns before and after ad/de-sorption-cycles measurement S13

Supplementary Fig. 12 PXRD patterns of lcp phase upon heating under atmospheric condition S14

Supplementary Fig. 13 PXRD patterns of lcp phase under vacuum S15

Supplementary Fig. 14 PXRD patterns of lcp phase upon heating in a vacuum condition S16

Supplementary Fig. 15 The full PXRD patterns during the water ad/de-sorption process S17

Supplementary Fig. 16 PXRD patterns of sample maintained at 433 K under vacuum S18

Supplementary Fig. 17 PXRD patterns of nqp phase in saturated steam and liquid water S19

Supplementary Fig. 18 Activation-method dependent water-adsorption S19

Supplementary Fig. 19 Micro-Raman spectra of raw materials S20

Supplementary Fig. 20 Micro-Raman spectra of partially dehydrated and nqp single crystals S21

Supplementary Fig. 21 Experimental setup for Micro-Raman spectroscopy S21

Supplementary Fig. 22 Temperature-dependence $\chi_M T$ curves of partially dehydrated samples S22

Supplementary Fig. 23 DSC curve of **1**·9/2H_2O S23

Supplementary Fig. 24 Variable-temperature synchrotron PXRD patterns of **1**·9/2H_2O S23

Supplementary Fig. 25 Rietveld refinement pattern of **1**·9/2H_2O at 100 K S24

Supplementary Fig. 26 DSC curve of **1**·4/3H_2O S24

Supplementary Fig. 27 Temperature-dependence $\chi_M T$ curves of **1**·4/3H_2O at different scan rates S25

Supplementary Fig. 28 The in-plane pedal rotations and directional shifts of Fe−N bonds S26

Supplementary Fig. 29 The variations in the pore configuration S27

Supplementary Fig. 30 The angular variations of 2D coordination network and the N-N-Fe angles S27

Supplementary Fig. 31 The interlayer molecular interactions of lcp phase and nqp phase S28

Supplementary Fig. 32 The relative slippage of 2D layers from lcp phase to nqp phase S28

Supplementary Fig. 33 The possible structural transition between nqp and lcp phases...................... S28

Supplementary Fig. 34 The energy diagram of water adsorption S29

Supplementary Fig. 35 The unit cells of **1**·5H_2O and **1**·4/3H_2O used for calculation S29

Supplementary Tables S30

Supplementary Table 1 Crystal data and structural refinements for **1**·9/2H_2O S30

Supplementary Table 2 Crystal data and structural refinements for **1**·4/3H_2O S31

Supplementary Table 3 Selected bond lengths and angles for **1**·9/2H_2O at different temperatures ... S32

Supplementary Table 4 Selected bond lengths and angles for **1**·4/3H_2O S32

Supplementary Table 5 Selected structural parameters for **1**·9/2H_2O and **1**·4/3H_2O at different temperatures S33

Supplementary Table 6 Selected parameters for prentrz ligands of **1**·$9/2H_2O$ at different temperatures S34

Supplementary Table 7 Selected parameters for prentrz ligands of **1**·$4/3H_2O$ at 100 K S34

Supplementary Table 8 Comparison between experimental and calculated lattice parameters S35

Supplementary Table 9 The Gibbs free energy of each structure with ZPE correction and entropy correction at 300 K during the water-adsorption-induced porous transformation S35

Supplementary References..S36

Supplementary Methods:

Synthesis of ligand (1*E*,2*E*)-3-phenyl-N-(4H-1,2,4-triazol-4-yl)prop-2-en-1-imine (prentrz). The ligand prentrz was synthesized by a modified literature procedure[1]. 4-NH_2-1,2,4-triazole (2.188 g, 25 mmol) and cinnamaldehyde (3.304 g, 25 mmol) was dissolved in 60 mL CH_3OH solution. The mixture solution was stirred and refluxed for six hours. The reaction solution was evaporated in the air atmosphere for two days, and then the white crystalline product of ligand was obtained and confirmed by single-crystal X-ray diffraction (yield: 2.963 g, ~ 59.8% based on cinnamaldehyde).

Differential scanning calorimetry (DSC). DSC measurements were recorded with PerkinElmer DSC 8000.

Thermogravimetric analyses (TGA). TGA measurements were recorded under a nitrogen flow (200 ml min^{-1}) on a Shimadzu DTG-60AH instrument in a temperature range of 293−775 K with rates of 0.25 K min^{-1} from 293 to 423 K, 0.5 K min^{-1} from 423 to 473 K and 2 K min^{-1} from 473 to 773 K.

Computational Details. The experiment obtained two crystal structures, one is nqp phase structure with 4 water molecules in the unit cell, denoted as **nqp-4w**; the other is lcp phase structure with 10 water molecules in the unit cell, denoted as **lcp-10w**. The optimized lcp phase structure contains 10 water molecules in the unit cell, which reflects the disorder state of guest water molecules in the pores. The number of water molecules, 9 or 10, has no influence on the procedure of adsorption-induced nqp–to–lcp lattice transformation. Here we use **-nw** to represent the number (n) of water molecules (w). These experimental results suggest that $[Fe^{II}(prentrz)_2Pd^{II}(CN)_4]$ (**1**) adsorbs 4 water molecules in the nqp phase and 10 water molecules in the lcp phase. To understand these results, we calculated the Gibbs energy changes ($\Delta G°$) for adsorption of H_2O molecules into **1** in both **nqp** and **lcp** phases. The chemical formula of the unit cell of **nqp-4w** is $[Fe_3Pd_3C_{78}H_{60}N_{36}]\cdot 4H_2O$, and the chemical formula of the unit cell of **lcp-10w** is $[Fe_2Pd_2C_{52}H_{40}N_{24}]\cdot 10H_2O$. Our calculation is performed on the unit cell of **nqp** and **lcp** structures, therefore, to make the energetics comparable, we balanced the number of atoms via multiplying the energetics of **nqp** structures by 2 and multiplying the energetics of **lcp** structures

by 3. For example, the energy difference between **nqp-0w** and **lcp-0w** is calculated as $\Delta E = 2\cdot E_{\text{nqp-0w}} - 3\cdot E_{\text{lcp-0w}}$, is −2.00 eV, as shown in Fig.6 and Supplementary Fig. 34.

The adsorption energy was calculated using the density functional theory (DFT) method as implemented in the Vienna ab initio simulation package (VASP 5.4.4)[2]. The generalized gradient approximation (GGA) of Perdew-Burke-Ernzerhof functional[3] with Grimme's semi-empirical "D3" dispersion term (PBE-D3) were chosen to describe electron-ion interactions[4,5]. DFT + U method was used to account for the strong correlation for the transition metals, where the U parameter of 4.0 eV and 2.0 eV was used for Fe and Pd, respectively[6,7]. The plane wave basis sets with an energy cutoff of 520 eV were used to describe valence electrons and the projector-augmented-wave pseudopotentials were used to describe core electrons. An energy convergence of 1×10^{-5} eV/atom and a force convergence of 0.03 eV/Å was used. The Brillouin zone was sampled by a Γ-point in these calculations. All the atoms are relaxed during the structural optimization. As shown in Supplementary Table 8, this method reproduces the experimental lattice parameters of **nqp-4w** and **lcp-10w**. When searching for the most stable structures of **nqp-2w**, we considered four types: type I, denoted as **nqp-2w-L**, **L** for left; type II, denoted as **nqp-2w-R**, **R** for right; type III, denoted as **nqp-2w-W**, **W** for weak; and type IV, denoted as **nqp-2w-S**, **S** for strong (shown in Supplementary Fig. 34).

The binding energies (BE) of water with **nqp** and **lcp** were calculated with Supplementary equation 1;

$$BE = E(\mathbf{1}\cdot nH_2O)\text{-}E(\mathbf{1})\text{-}nE(H_2O) \qquad \text{(Supplementary equation 1)}$$

where $E(\mathbf{1}\cdot nH_2O)$ is the total energy of **1** in **lcp** or **nqp** phases with n guest molecules (H_2O) per unit cell, $E(\mathbf{1})$ is the total energies of empty **1** in **nqp** or **lcp** structure, and $E(H_2O)$ is the energy of one free guest molecule (H_2O).

To calculate the Gibbs energy change (ΔG), we calculated the vibrational energy of H_2O molecule in free and adsorbed geometries. The ΔG for adsorption of n H_2O molecule is calculated by Supplementary equation 2;

$$\Delta G = BE + \Delta\text{ZPE} + \Delta E^{\text{thermal}} - \text{n}RT - T\Delta S \qquad \text{(Supplementary equation 2)}$$

where ΔZPE is the zero-point vibrational energy, $\Delta E^{\text{thermal}}$ is thermal energy, nRT is the pV term according to ideal gas approximation, and ΔS is the entropy change by adsorption of n guest molecules. We used VASPkit to obtain above values.[8]

Supplementary Figures:

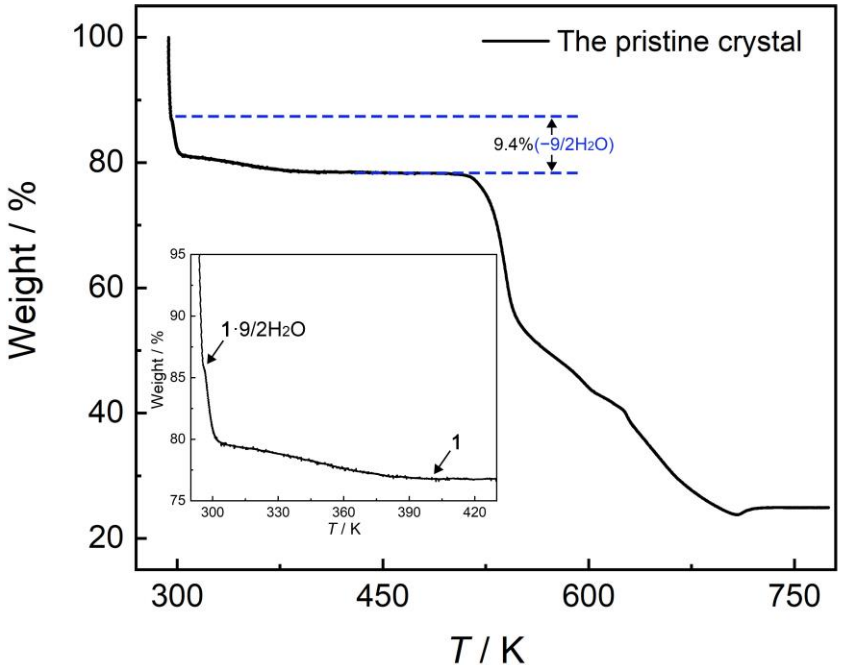

Supplementary Fig. 1 Thermogravimetric analysis of the pristine crystal (**1**·*x*MeOH·*y*H_2O). Thermogravimetric analysis of the pristine crystal. The pristine crystals prepared from MeOH/H_2O mixture contain both H_2O and MeOH molecules that can be partially exchanged when the sample is exposed in the air. The first step weight loss in the TG curve suggests a fast exchange and removal of MeOH guests in the room temperature, and the second step weight loss corresponds to the **1**·9/2H_2O to **1**·4/3H_2O transformation, and the third step weight loss indicates the **1**·4/3H_2O to **1** transformation. Notably, although the TG measurement suggests the guest H_2O molecules were removed at ca. 400 K, a small part of **1**·9/2H_2O (lcp phase structure) remains according to PXRD and vapor adsorption isotherm measurements.

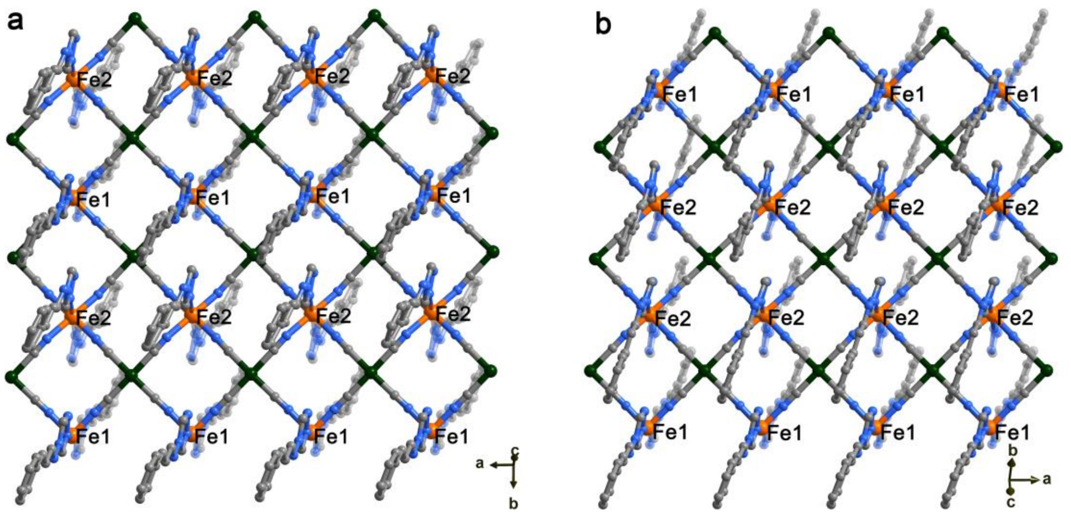

Supplementary Fig. 2 Layer structure of **1**·9/2H_2O and **1**·4/3H_2O. The equatorial positions of both Fe1 and Fe2 are coordinated by four nitrogen atoms from four $[Pd(CN)_4]^{2-}$ to connect the 2D coordination network, and the axial positions are occupied by two nitrogen atoms from two prentrz ligands. (**a**) **1**·9/2H_2O. (**b**) **1**·4/3H_2O.

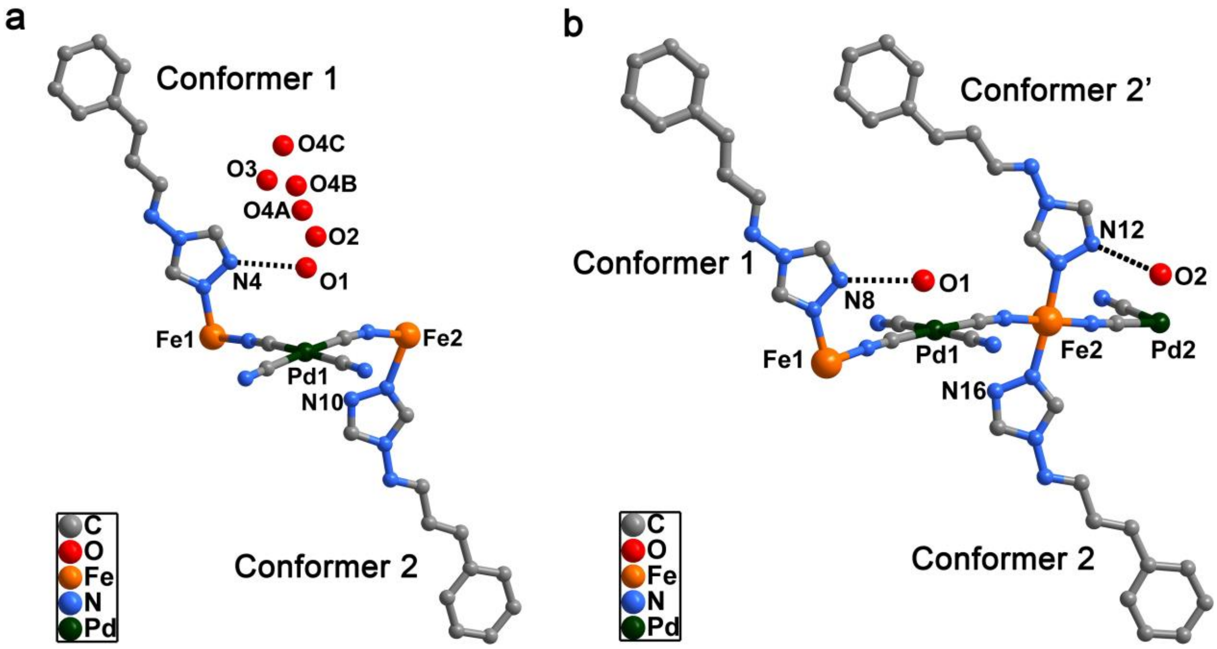

Supplementary Fig. 3 The asymmetric unit of **1**·9/2H_2O (**a**) and **1**·4/3H_2O (**b**).

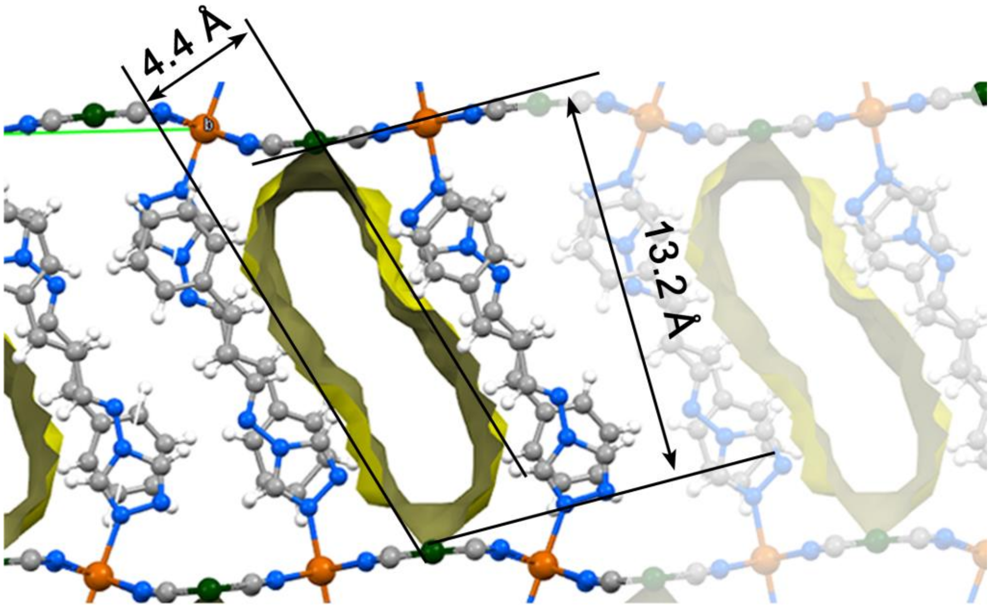

Supplementary Fig. 4 Pore structure and void space of **1**·9/2H_2O illustrated by Connolly surface using a probe of diameter of 1 Å along *a*-axis.

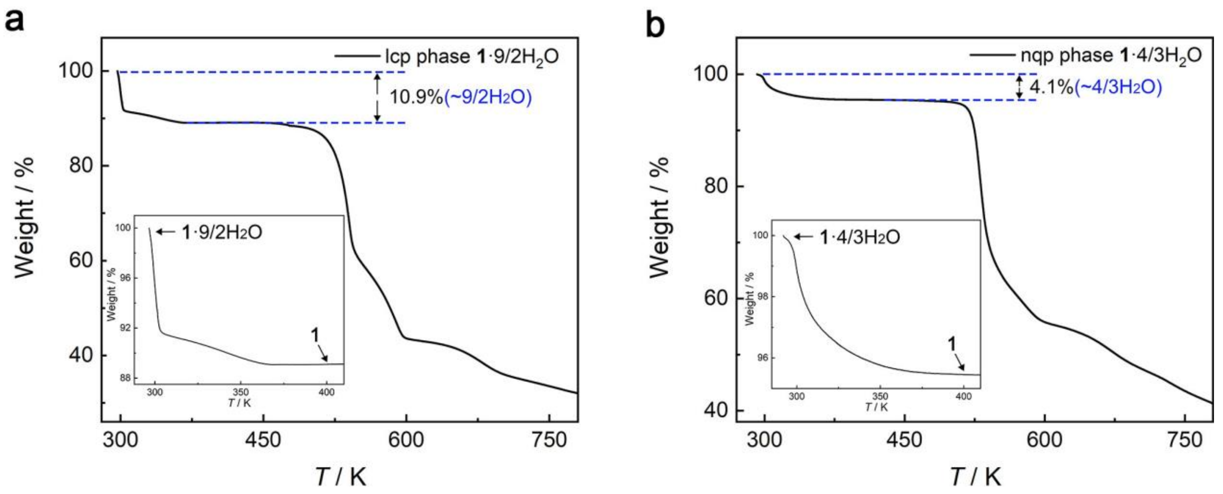

Supplementary Fig. 5 Thermogravimetric analysis (TGA) of lcp phase **1**·9/2H_2O (**a**) and nqp phase **1**·4/3H_2O (**b**). The anhydrous sample is stable up to ca. 503 K.

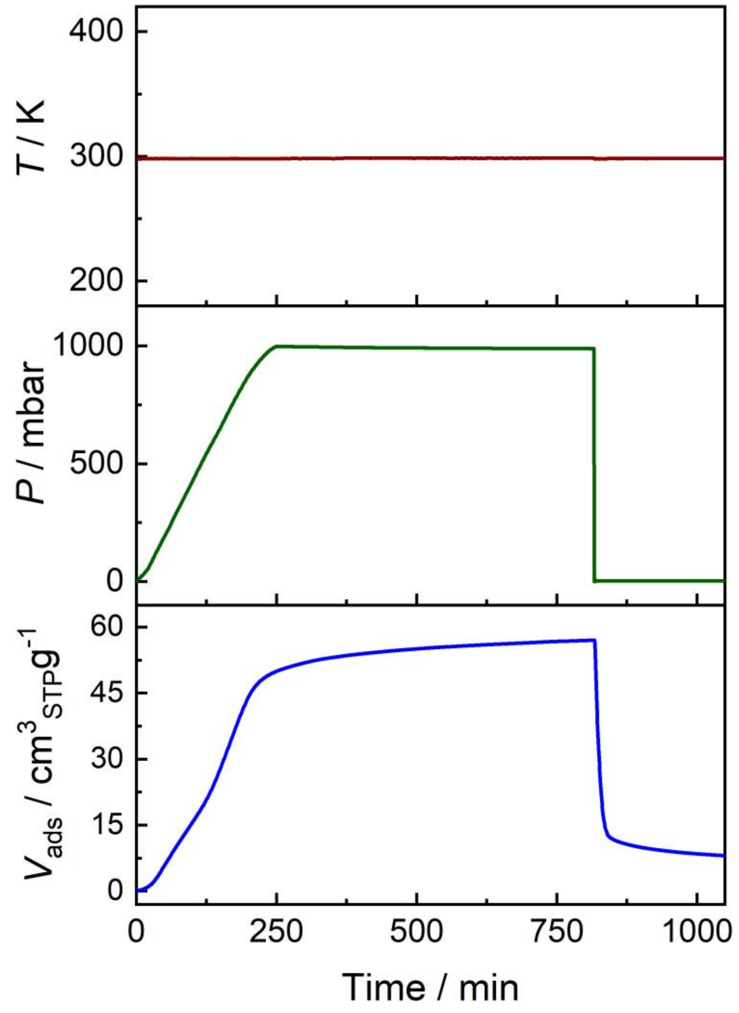

Supplementary Fig. 6 CO_2 adsorption of lcp phase **1**·9/2H_2O activated under mild activated conditions (303 K under vacuum for 5 h).

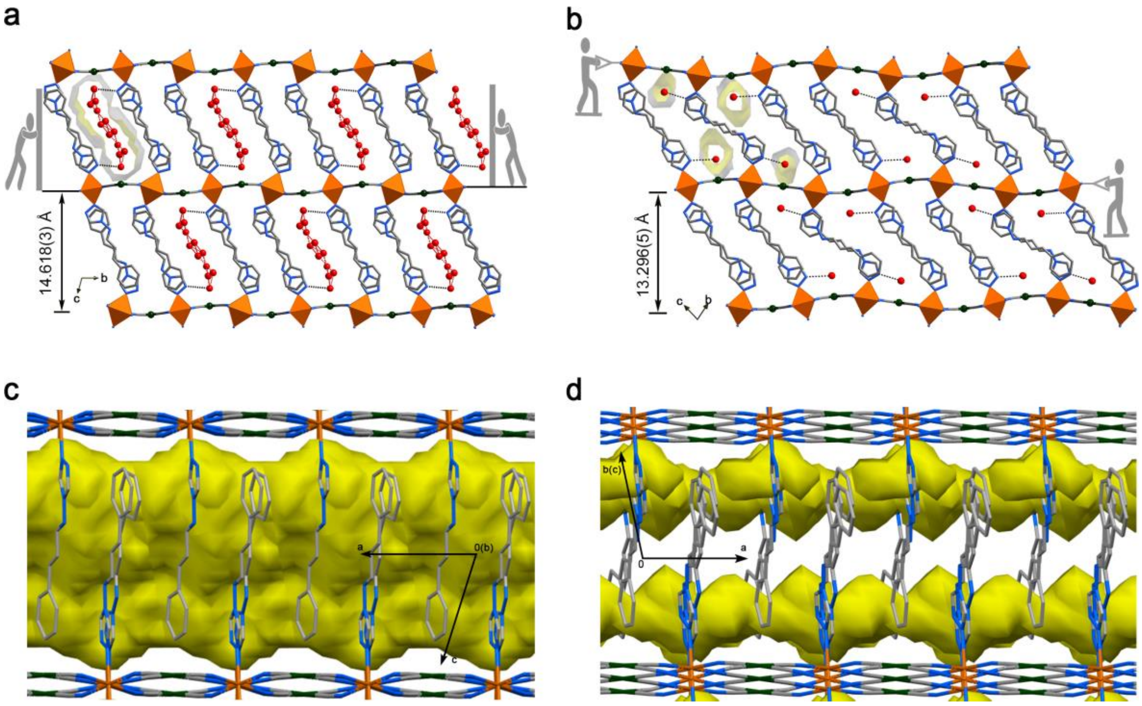

Supplementary Fig. 7 Crystal structures and pore shapes of lcp phase **1**·9/2H_2O and nqp phase **1**·4/3H_2O. **a** 3D framework of **1**·9/2H_2O with an interlayer distance of 14.618 Å. **b** 3D framework of **1**·4/3H_2O with an interlayer distance of 13.296 Å. **c** The large channel-type pores of **1**·9/2H_2O extending along the crystallographic *a*-axis. **d** The narrow quasi-discrete pore of **1**·4/3H_2O along the *a*-axis.

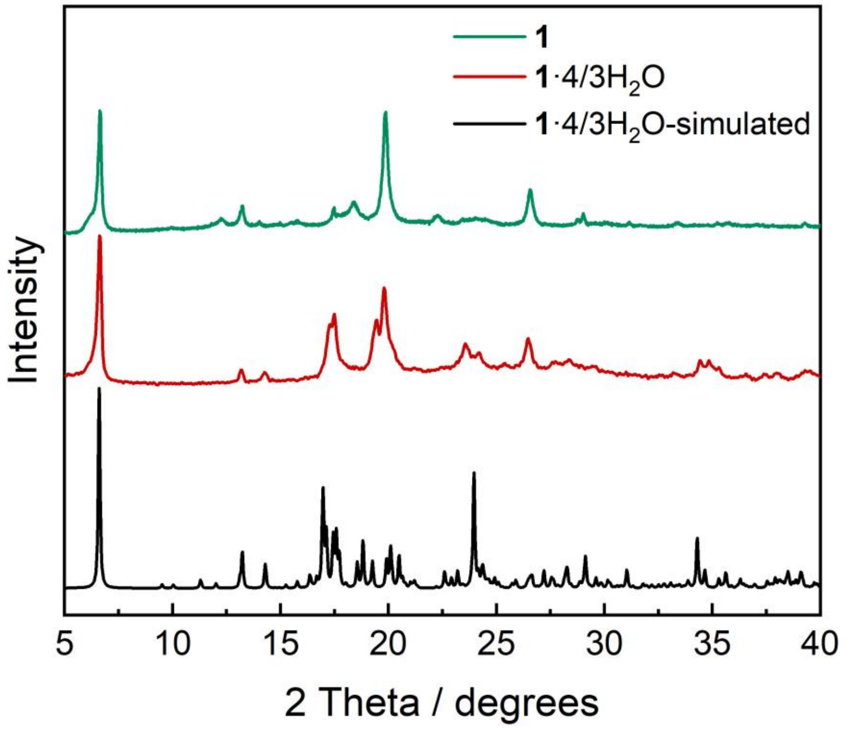

Supplementary Fig. 8 *In situ* PXRD patterns of **1** upon heating the dried sample at 433 K for 48h under vacuum (Green, the heating rate of 5 K min^{-1}) compared with that of **1**·4/3H_2O (Red). The PXRD data of **1** was collected at room temperature after activated treatment.

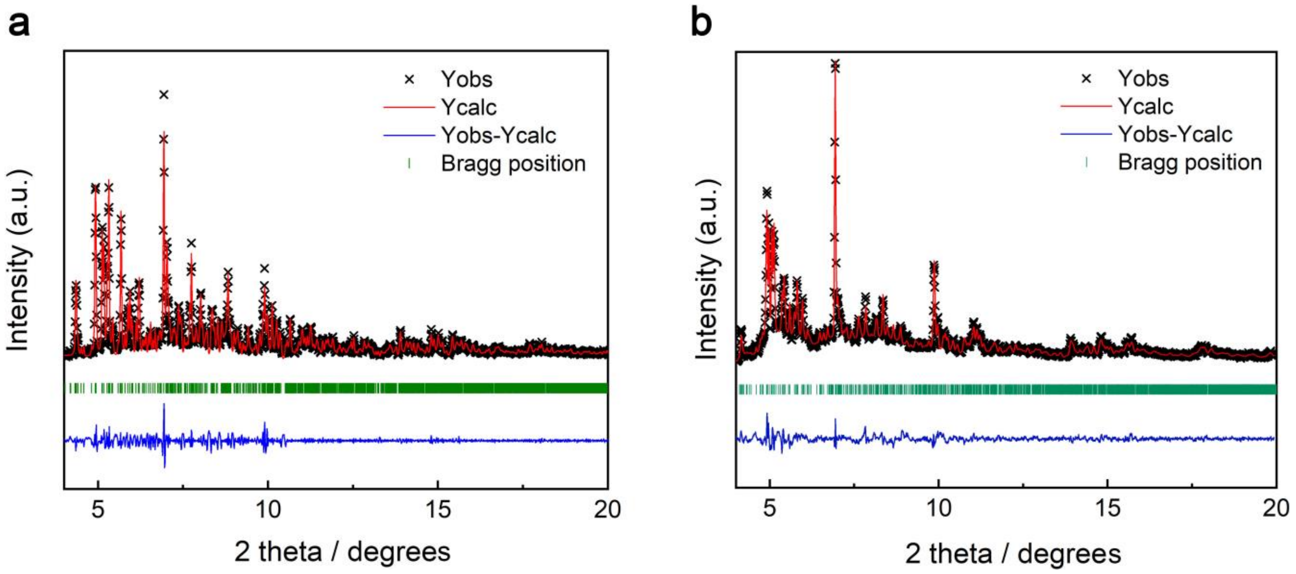

Supplementary Fig. 9 Rietveld refinement pattern and PXRD pattern of lcp phase **1**·9/2H_2O and nqp phase **1**·4/3H_2O at 250 K. **a** The reduced unit cell parameters of a = 7.456(6) Å, b = 14.706(5) Å, c = 15.381(6) Å, α = 104.3601(1)°, β = 99.863(2)°, γ = 90.274(2)°, V = 1607.93(8) Å^3 are in agreement with those of single crystal analyses of **1**·9/2H_2O at 250 K. Rp = 2.07%, Rwp = 3.26%. **b** The reduced unit cell parameters of a = 7.4590(4) Å, b = 16.304(3) Å, c = 19.238(8) Å, α = 76.825(2)°, β = 80.204(1)°, γ = 78.522(3)°, V = 2213.23(6) Å^3 are in agreement with those of single crystal analyses of **1**·4/3H_2O at 250 K. Rp = 1.71%, Rwp = 2.37%.

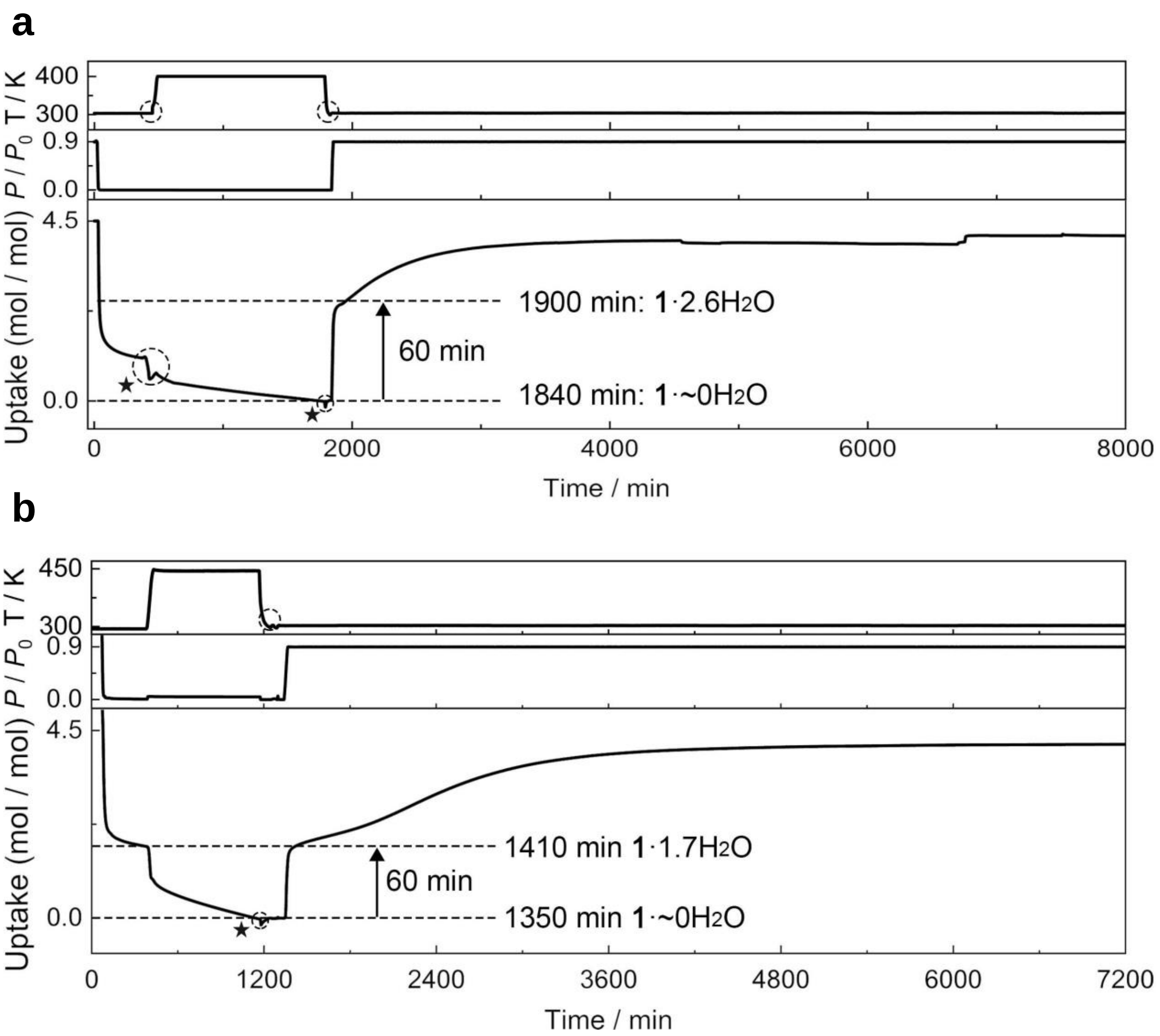

Supplementary Fig. 10 The water adsorption-desorption measurements of the sample activated at different temperatures. **a** The sample activated at 400 K under vacuum for 22 h. **b** The sample activated at 433 K under vacuum for 14 h. Although the measurements indicate that almost all water guests are removed upon heating at 400 K, the uptake of ca. 2.6 H_2O per Fe^{II} for the sample activated at 400 K for 22 h is larger than that of ca. 1.7 H_2O per Fe^{II} for the smaple activated at 433 K for 14 h, suggesting more crystals of partially dehydrated framework survived in the former, and this reslut is consistent with the PXRD measurements. Star marker: The abnormal drops of "Uptake" line is ascribed to the change of heating source

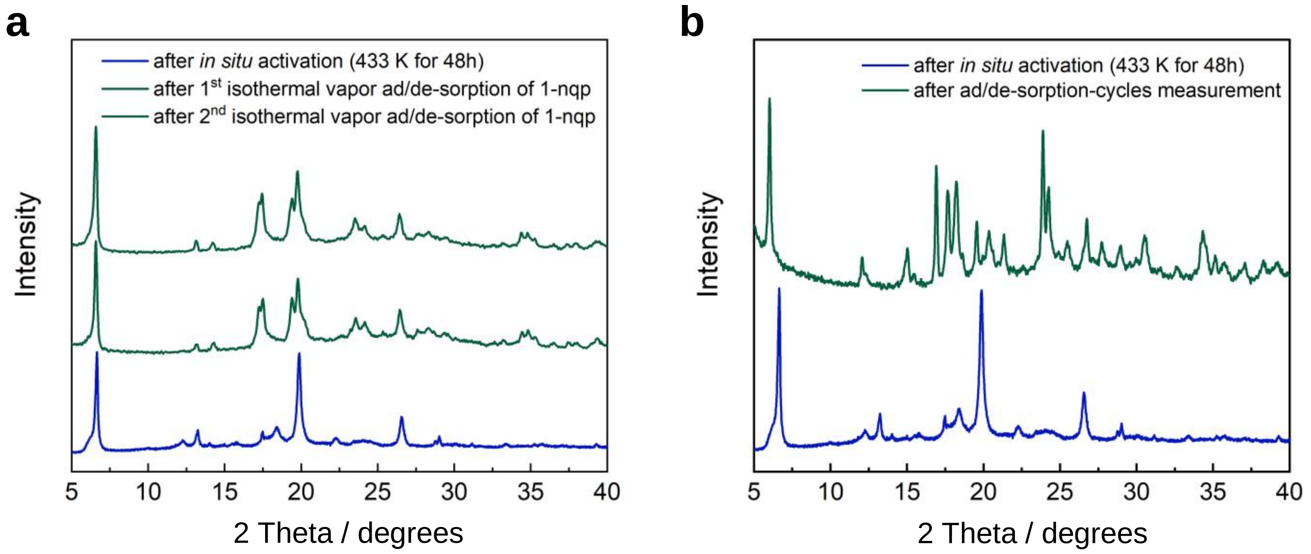

Supplementary Fig. 11 PXRD patterns of samples after *in situ* activation (Blue) and after two adsorption isotherm experiments and the continuous adsorption-desorption-cycles measurement (Green). **a** The PXRD pattern of sample after two isothermal vapor ad/de-sorption (in the Fig. 3A up) of the sample activated at 433 K under vacuum for 48 h. **b** The PXRD pattern of sample after a continuous adsorption-desorption-cycles measurement (in the Fig. 3B, including a long-time heating at 433 K under vacuum and a mild condition at 303 K under vacuum) reveals that the crystallinity and framework of this sample can be revived by rehydration.

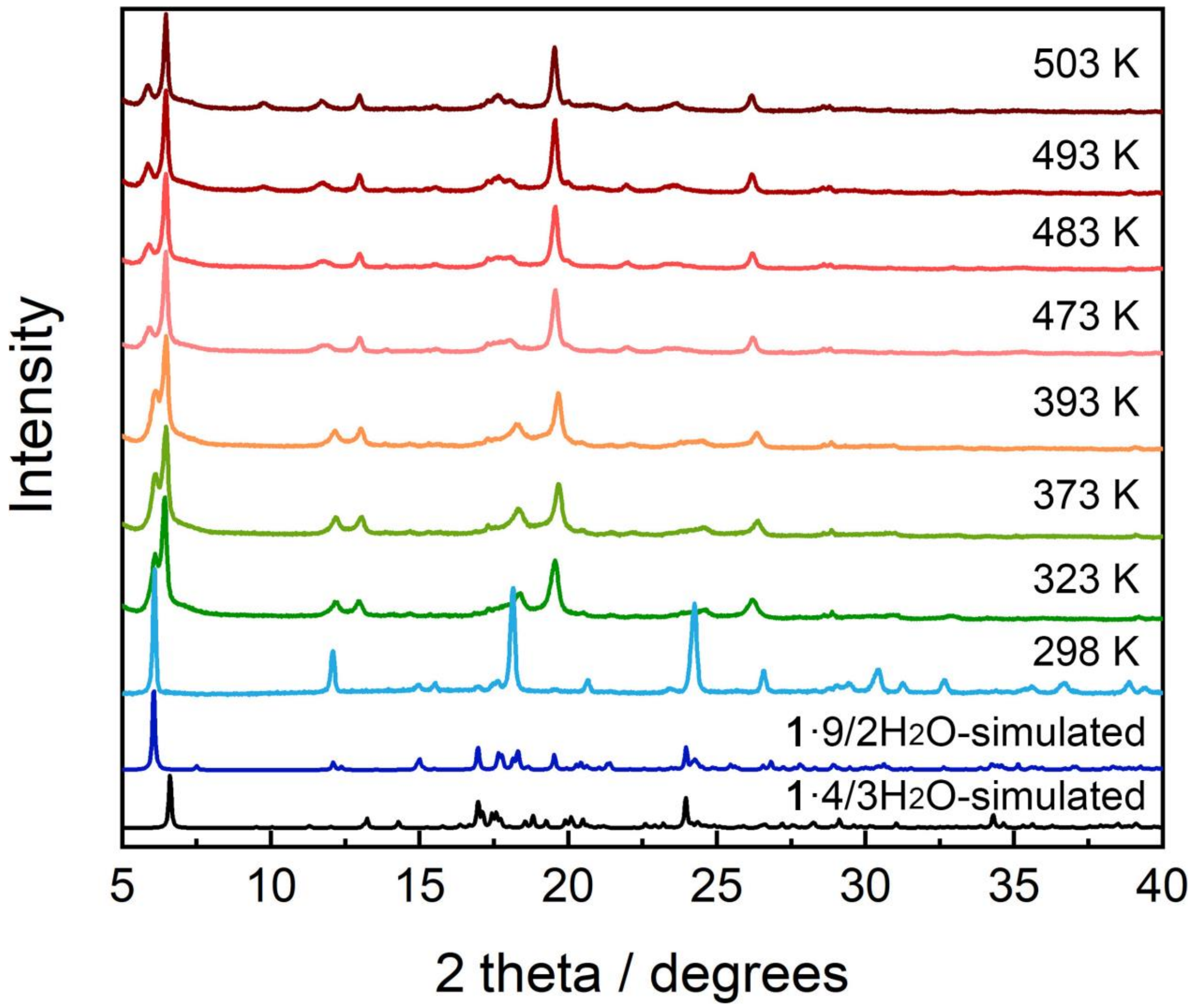

Supplementary Fig. 12 PXRD patterns of lcp phase **1**·9/2H_2O upon heating under atmospheric condition. The peaks denoting the nqp phase appeared when the lcp phase was heated at 323 K for 1 min, and the partially dehydrated residue persisted to 503 K. The PXRD data at each temperature were collected after a holding time of 10 min. The PXRD measurements were performed on finely grounded polycrystals. The strong preferred orientation in the powder diffraction data is due to the structural nature of 2D coordination network of the samples.

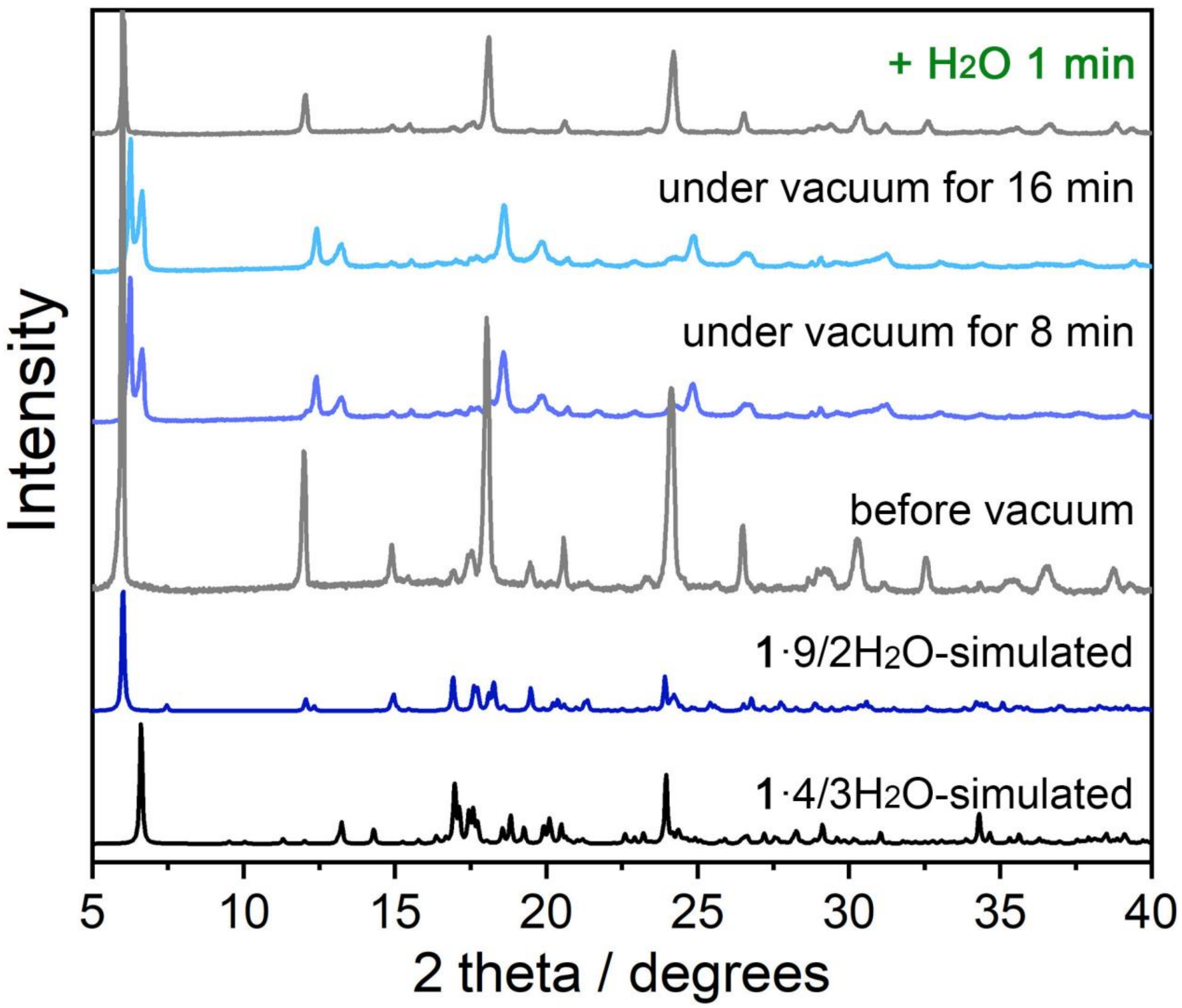

Supplementary Fig. 13 PXRD patterns of lcp phase **1**·9/2H_2O under vacuum. The peaks denoting the nqp phase immediately appeared when the sample of lcp phase **1**·9/2H_2O was placed under vacuum. The lcp phase was recovered instantly by spraying water mist on the partially dehydrated sample.

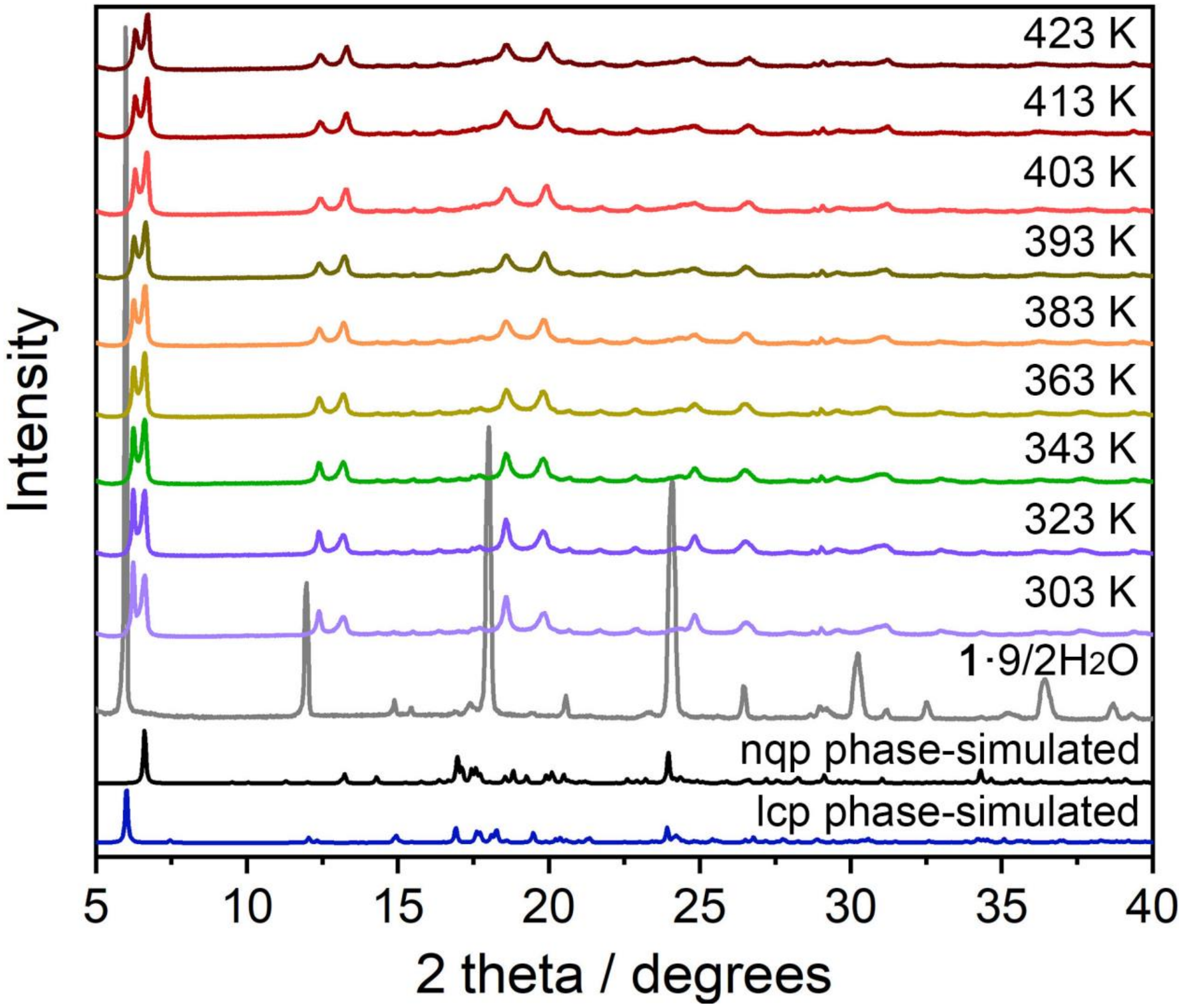

Supplementary Fig. 14 PXRD patterns of lcp phase **1**·9/2H_2O upon heating in a vacuum condition. The PXRD data at each temperature were collected after a holding time of 10 min.

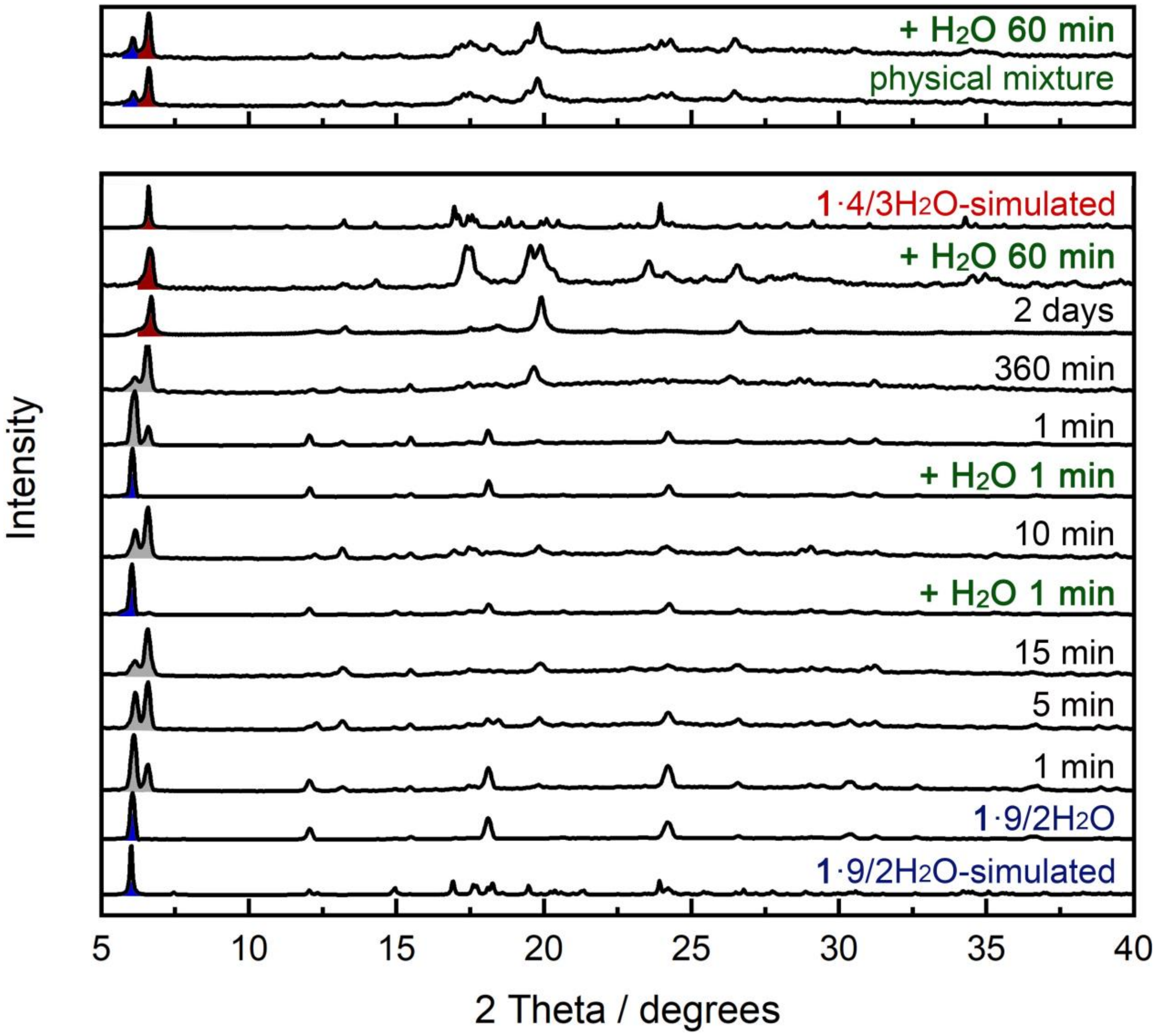

Supplementary Fig. 15 The full PXRD patterns during the water desorption-adsorption process. The sample was dehydrated rapidly upon heating at 393 K and the backward water adsorption was performed by spraying water mist on the sample. The partially dehydrated sample recovered to the lcp phase instantly, while the completely dehydrated sample **1** prepared from long-time heating (433 K) under vacuum can rehydrate to nqp phase **1**·4/3H_2O within 60 min and cannot recover to lcp phase smoothly upon water adsorption. The all data were collected at room temperature after different activated treatment. The PXRD patterns of the physical mixture of two pure phases did not show any significant change after spraying the water mist for 60 min. The gray, blue and red peaks denote the character peaks of structures of partially dehydrated, lcp and nqp frameworks, respectively.

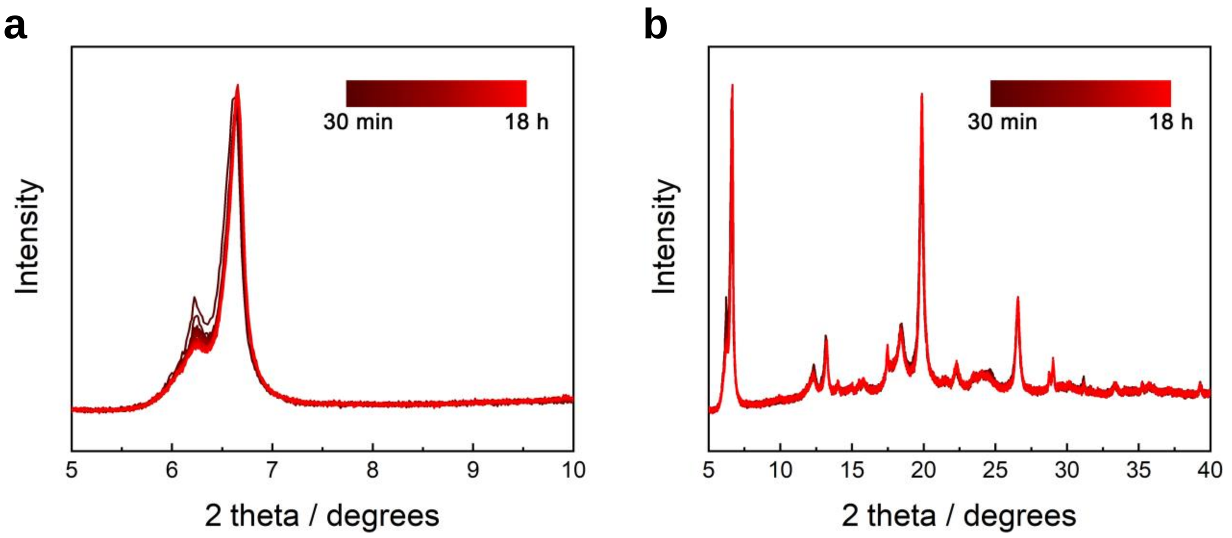

Supplementary Fig. 16 Local (**a**) and total (**b**) high-temperature PXRD patterns of sample maintained at 433 K under vacuum. The sample still has partially dehydrated residue even after heating at 433 K for 18 h.

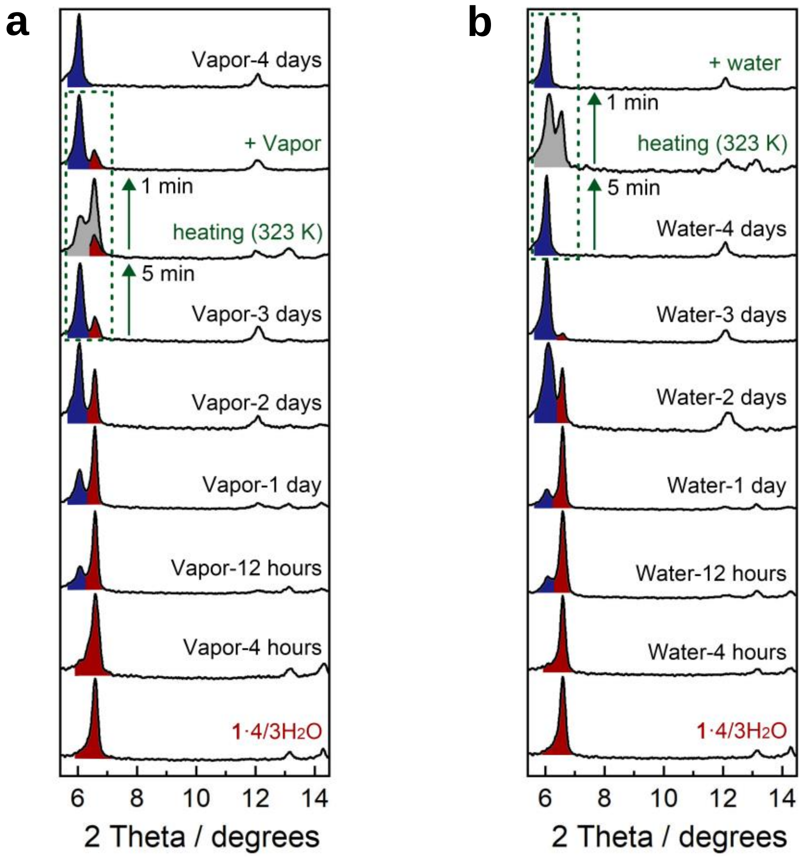

Supplementary Fig. 17 PXRD patterns of nqp phase **1**·4/3H_2O in saturated steam (**a**) and liquid water (**b**). The lcp phase **1**·9/2H_2O can be recovered by leaving the nqp phase **1**·4/3H_2O in saturated steam or water for *ca.* four days, while the partially dehydrated sample changes to lcp phase **1**·9/2H_2O within 1 minute, suggesting the different structural flexibility between the partially dehydrated and nqp samples. The all data were collected at room temperature after different activated treatment.

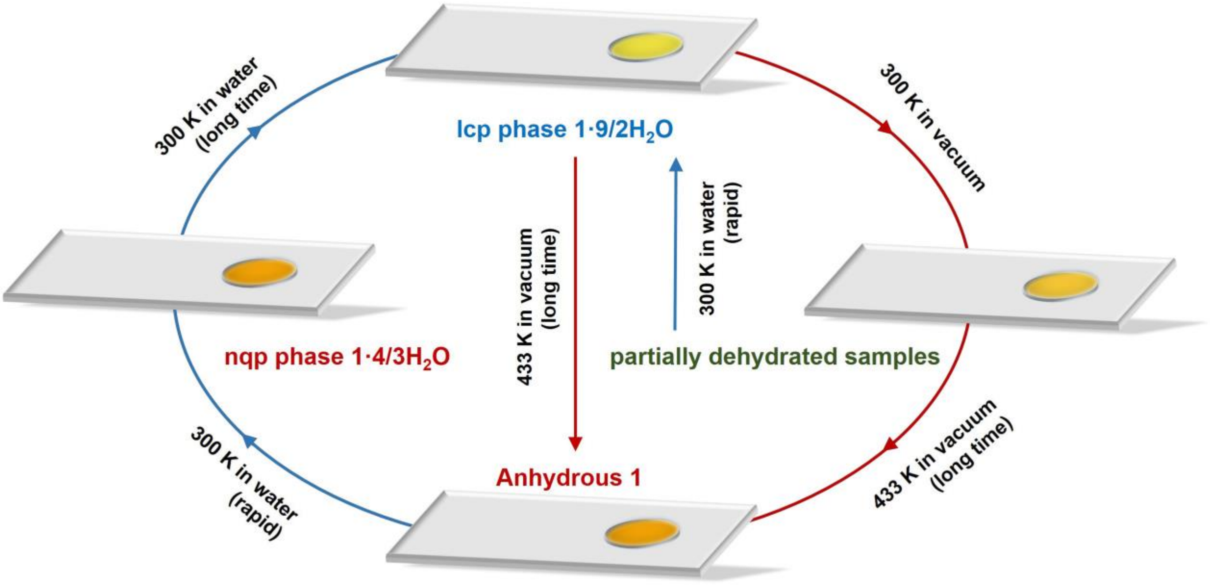

Supplementary Fig. 18 Activation-method dependent water-adsorption (Red lines: desorption; Blue lines: adsorption).

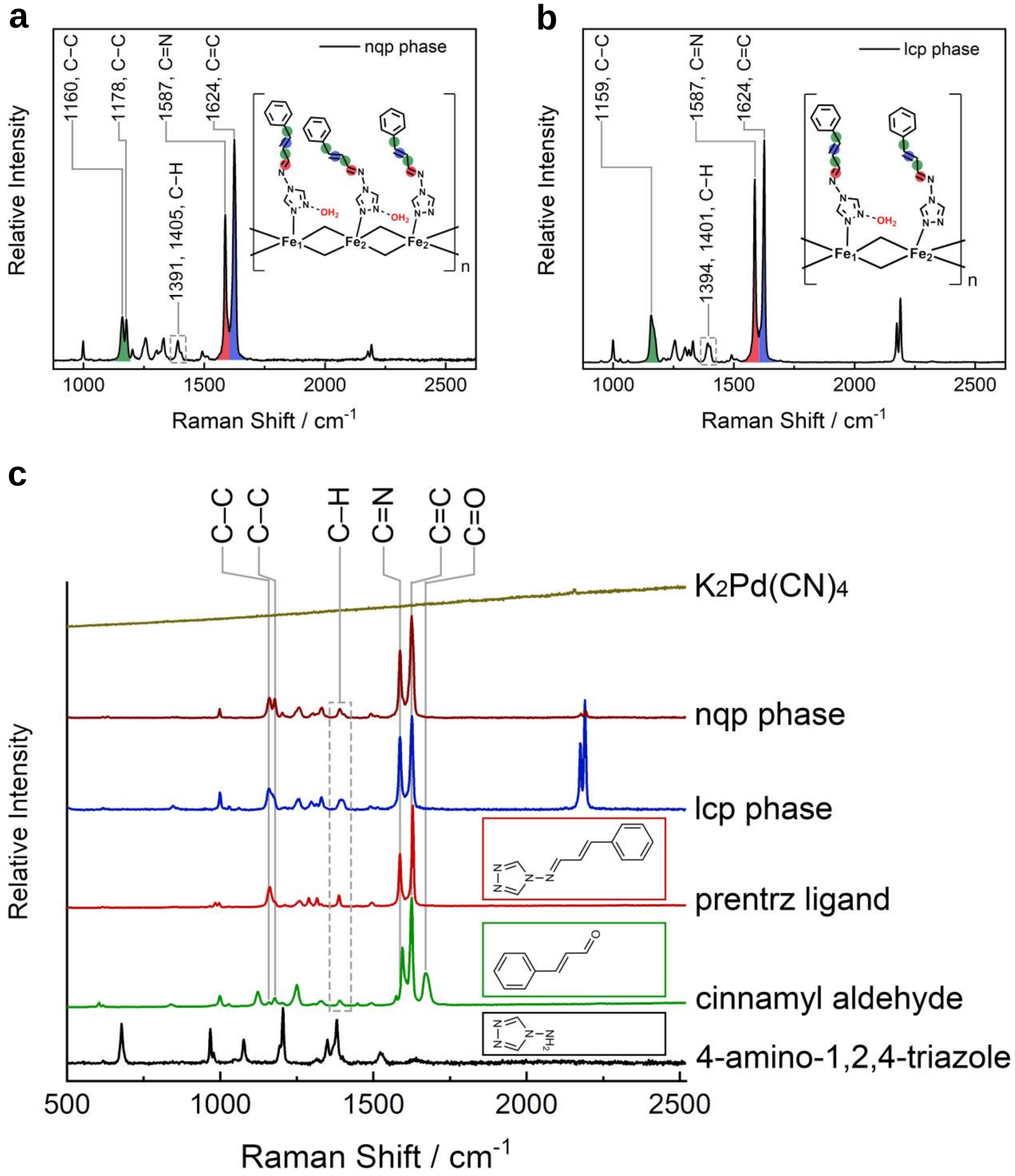

Supplementary Fig. 19 Micro-Raman spectra of nqp phase (**a**), lcp phase (**b**) and reactants (**c**). In the Supplementary Fig. 19c, the attribution of bands can be determined by comparison between reactants (4-amino-1,2,4-triazole, cinnamyl aldehyde, prentrz ligand and $K_2Pd(CN)_4$), lcp phase and nqp phase.

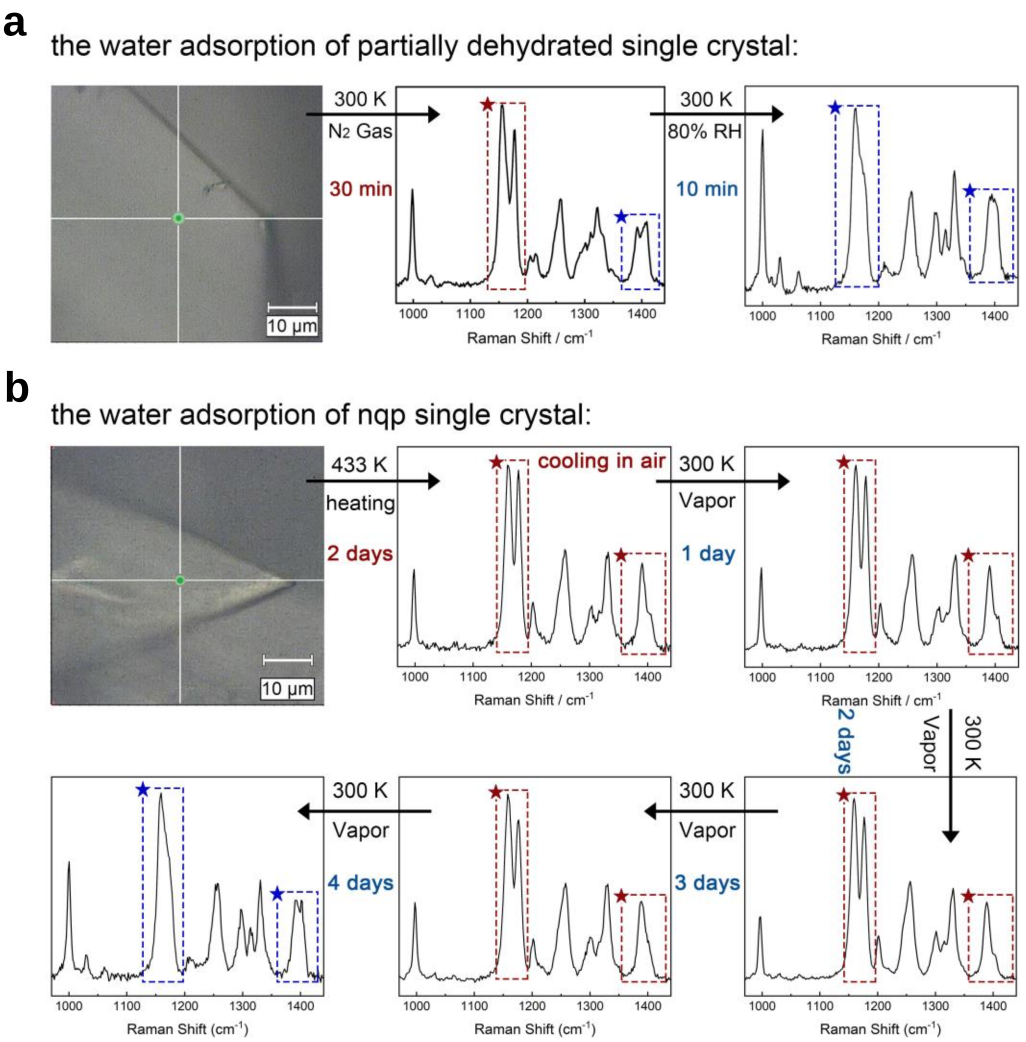

Supplementary Fig. 20 Micro-Raman spectra of partially dehydrated (**a**) and nqp single crystals (**b**) before and after water adsorption. The bands framed by dotted box represent lcp (blue) and nqp (red) structures.

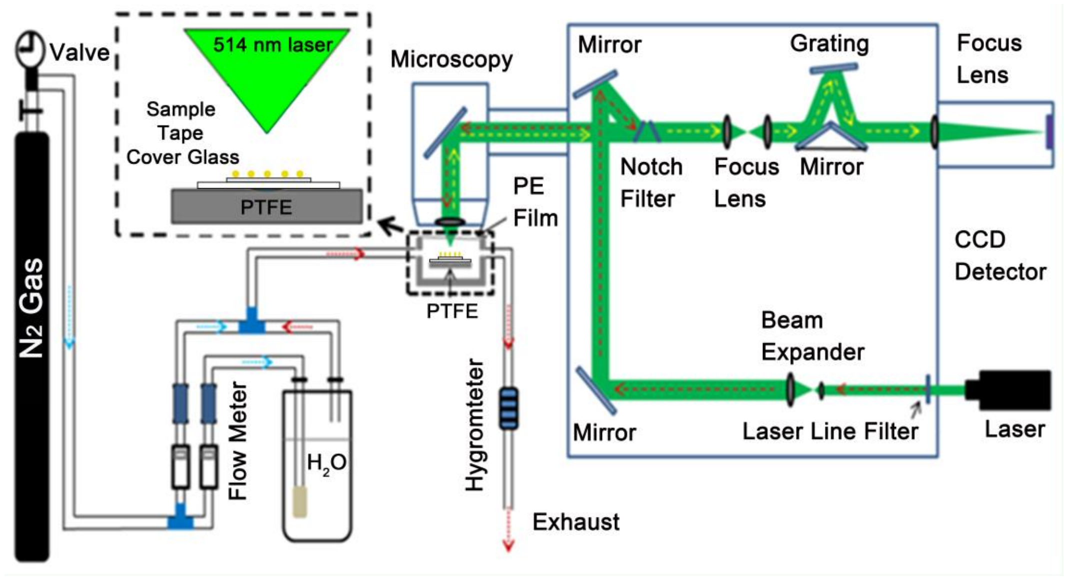

Supplementary Fig. 21 Experimental setup for Micro-Raman spectroscopy with different the different ambience.

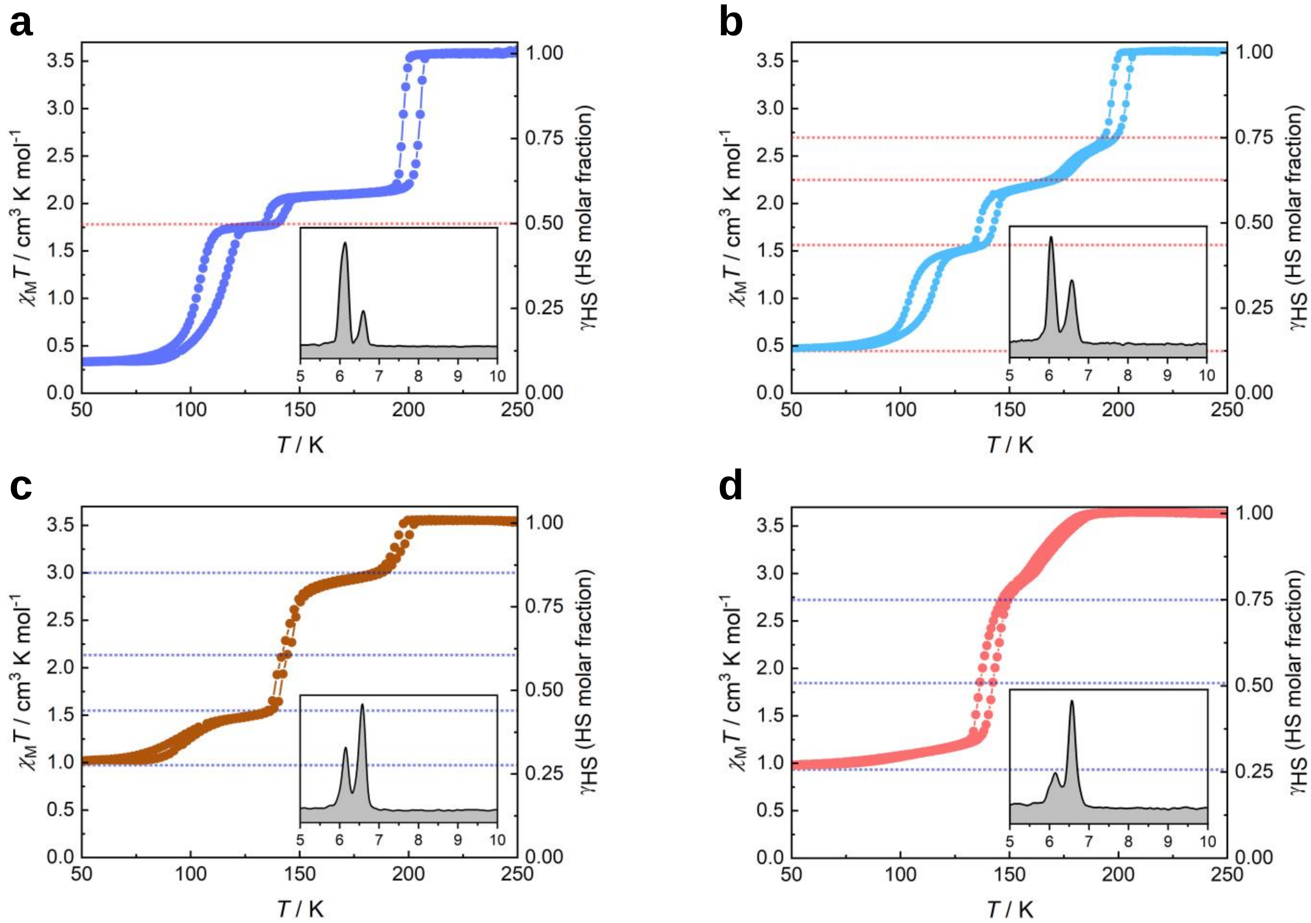

Supplementary Fig. 22 Temperature dependence of the $\chi_M T$ curves of partially dehydrated samples. The SCO of the partially dehydrated samples exhibited a multi-step spin transition in which the degree of magnetic switching can be adjusted by controlling the water molecules losing (**a**~**d**). The corresponding state of each sample was characterized by PXRD.

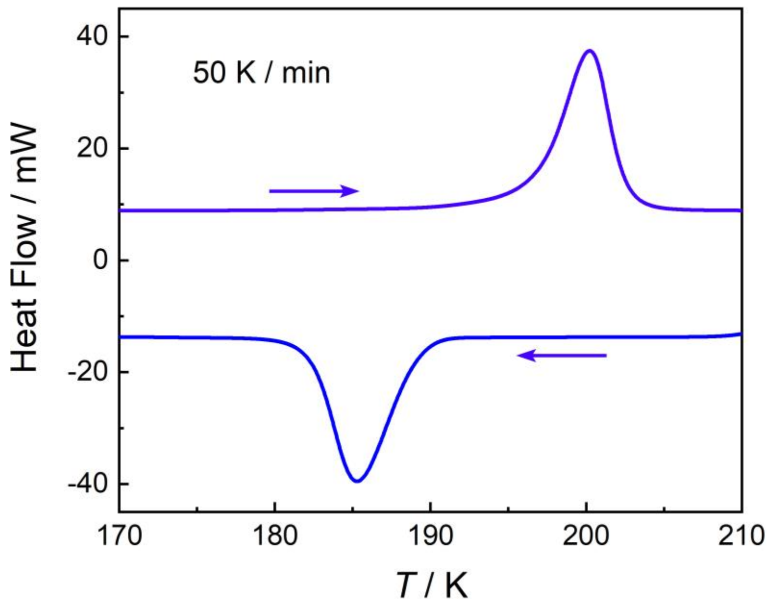

Supplementary Fig. 23 DSC curve of **1**·9/2H_2O. The distinct peaks with a substantial thermal hysteresis loop are consistent with the hysteretic spin transition observed at the high temperature range of **1**·9/2H_2O.

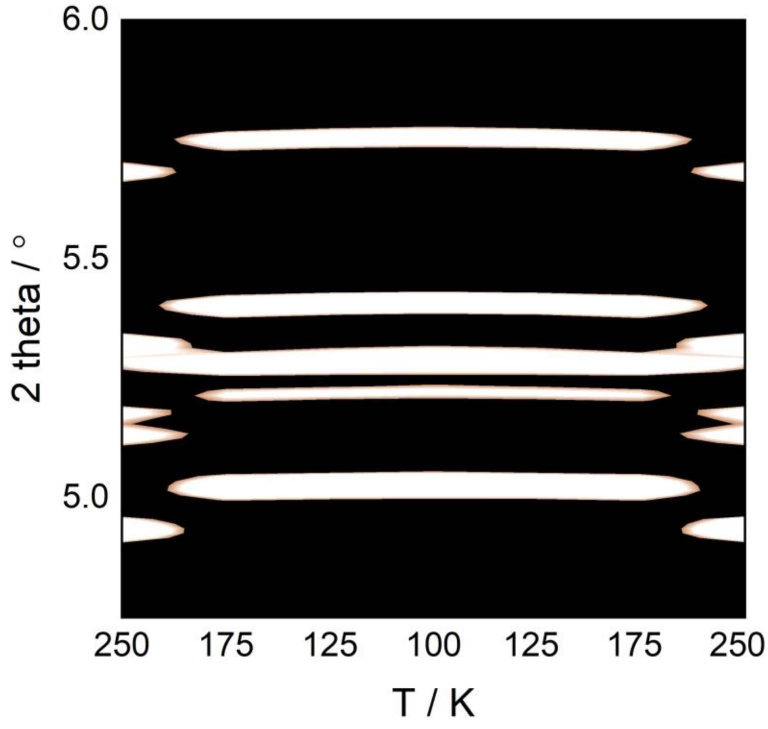

Supplementary Fig. 24 Variable-temperature synchrotron PXRD patterns of **1**·9/2H_2O. The shifts of Bragg peaks further verify the SCO transition at the high temperature range of **1**·9/2H_2O.

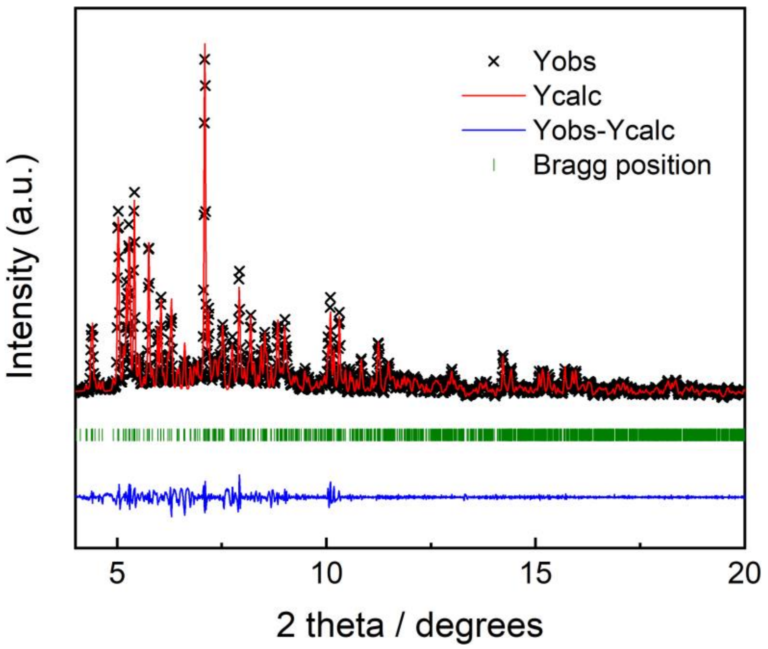

Supplementary Fig. 25 Rietveld refinement pattern of **1**·9/2H_2O at 100 K. The reduced unit cell parameters of a = 7.292(4) Å, b = 14.479(4) Å, c = 15.325(3) Å, α = 104.393(1)°, β = 100.002(1)°, γ = 90.295(2)°, V = 1486(6) Å^3, are in agreement with those of single crystal analyses. Rp = 1.75%, Rwp = 2.72%.

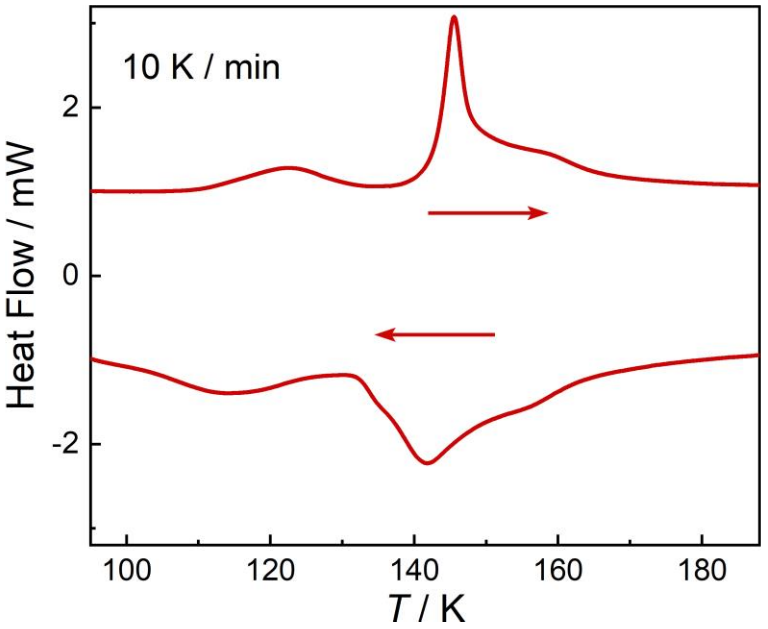

Supplementary Fig. 26 DSC curve of **1**·4/3H_2O. The two peaks identified in both heating and cooling processes are consistent with the two-step spin transition observed in the temperature-dependent magnetic susceptibility of **1**·4/3H_2O.

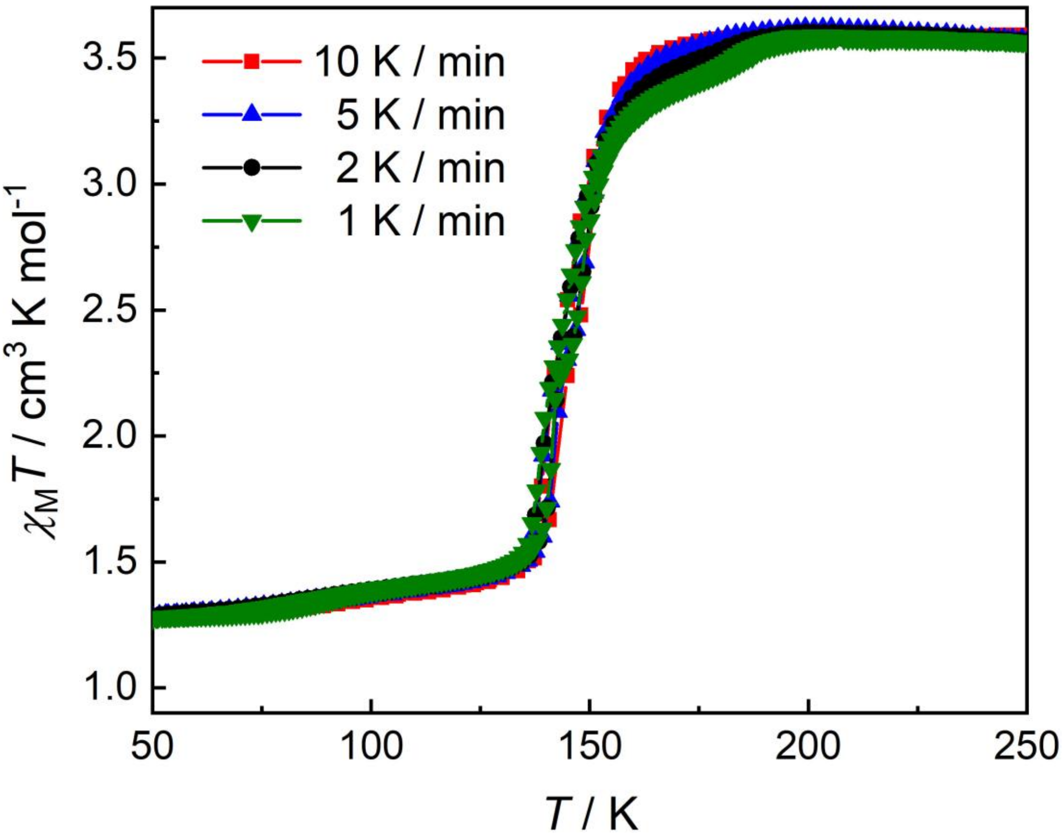

Supplementary Fig. 27 Temperature dependence of the $\chi_M T$ curves of nqp phase **1**·4/3H_2O at different scan rates (10, 5, 2 and 1 K min^{-1})

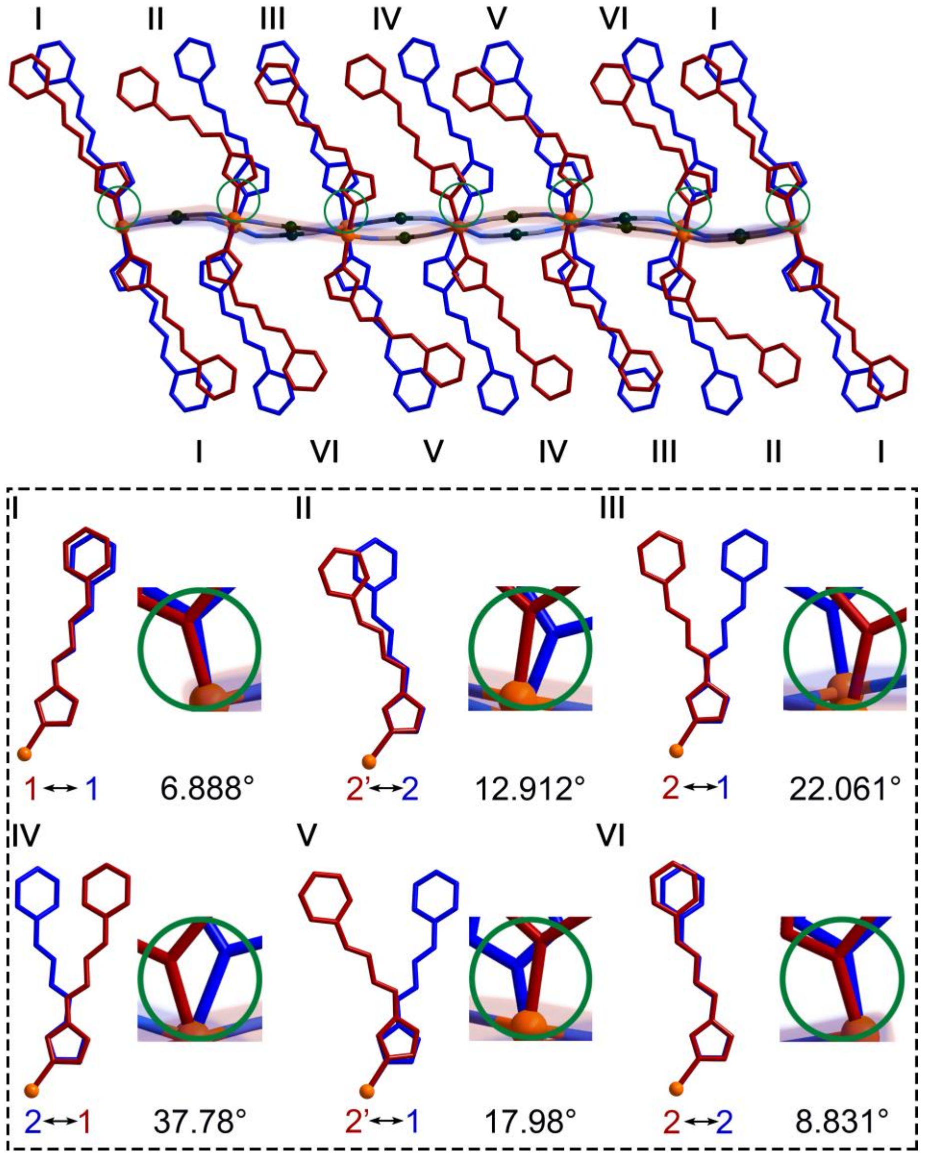

Supplementary Fig. 28 The detailed in-plane pedal rotations and directional shifts of Fe−N bonds of prentrz ligands. The ligands III, IV, and V perform pedal rotations around their N−N single bonds and the Fe−N coordination bonds of prentrz of II, III, IV, and V undergo remarkable shifts in their bond directions with reference to the 2D coordination layer. The directional shifts of Fe−N bonds and pedal rotations of ligands exert opposite influences on the ligand principal directions. Consequently, the ligands II and IV manifest most significant changes in their molecular principal directions, while those of ligands I, III, V, and VI are relatively small. The non-uniform reorientations of ligands repartition the lattice structure. The structures of **1**·9/2H_2O and **1**·4/3H_2O are drawn in blue and red, respectively.

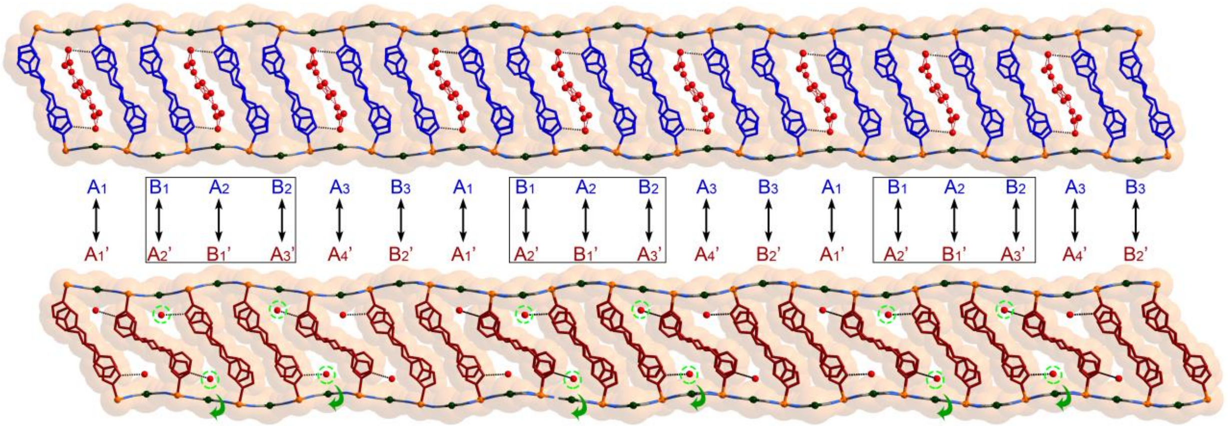

Supplementary Fig. 29 The variations in the pore configuration. The non-uniform directional shifts of axial ligands reversibly repartition the lattice structure, leading to a pore rearrangement from A'A'B'A'A'B' mode of npq phase **1**·4/3H_2O (bottom) to ABABAB mode of lcp phase **1**·9/2H_2O (top). The green arrows denote the occurrence of simultaneous pore opening and closing during nqp to lcp gate-opening transition.

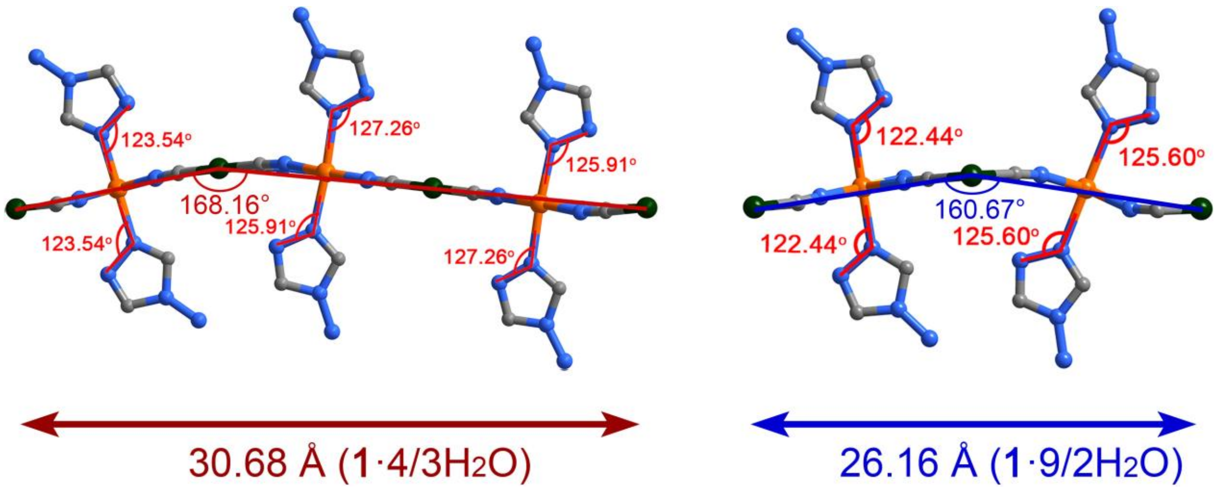

Supplementary Fig. 30 The angular variations of corrugated 2D coordination network and the N-N-Fe angles. During the water-adsorption induced nqp phase **1**·4/3H_2O to lcp phase **1**·9/2H_2O transition, the curvature varies from 168.16° to 160.67°, and the length of repeating unit changes from 30.68 Å to 26.16 Å, indicating the coordination layer undergoes a remarkable crumpling/unfolding motion. The angles of N-N-Fe of prentrz ligands also demonstrate substantial changes that contribute to the molecular reorientation in their principal axes.

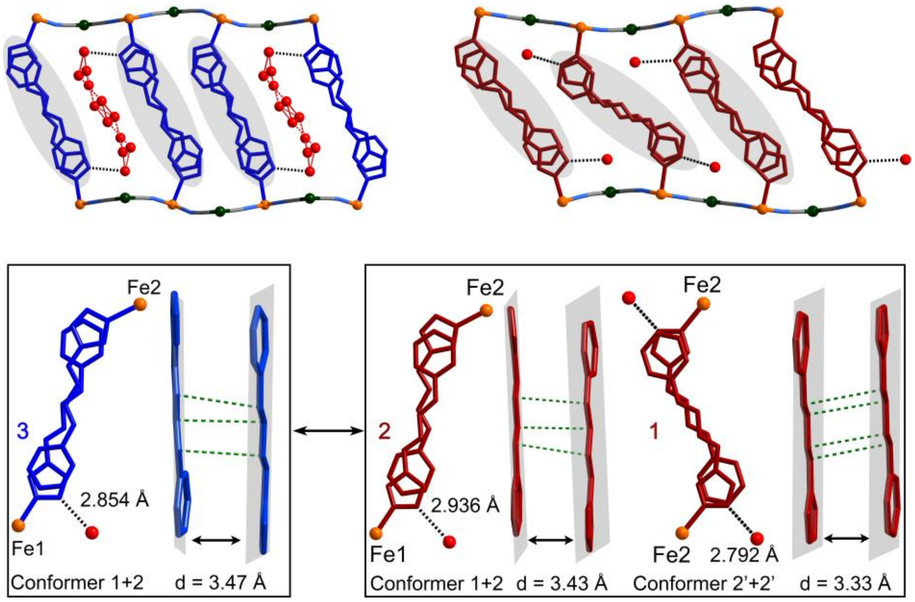

Supplementary Fig. 31 The interlayer molecular interactions of lcp phase **1**·9/2H_2O (left, in blue) and nqp phase **1**·4/3H_2O (right, in red). The 3D porous framework is constructed from 2D coordination networks via π–π interactions between the prentrz ligands in the adjacent layers.

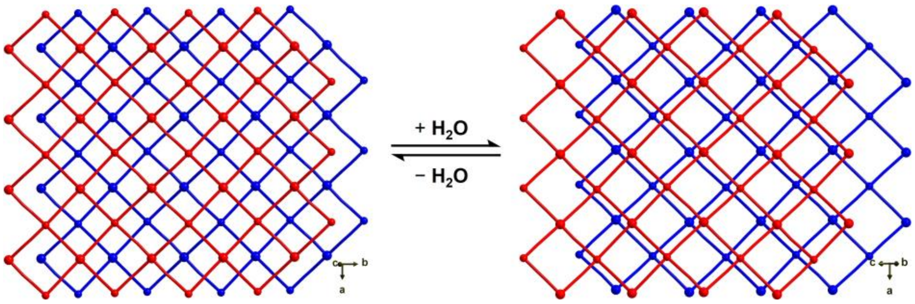

Supplementary Fig. 32 The relative slippage of 2D coordination layers from lcp phase **1**·9/2H_2O (left) to nqp phase **1**·4/3H_2O (right).

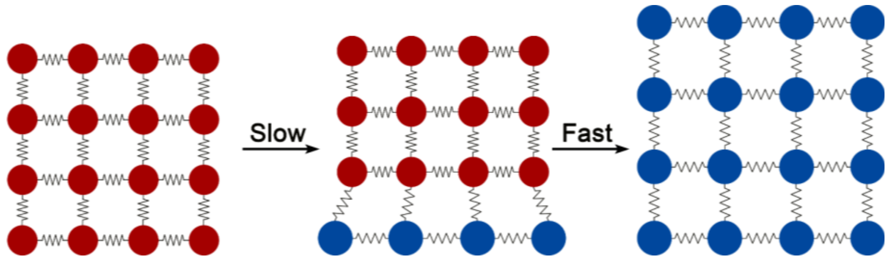

Supplementary Fig. 33 Scheme of the possible structural transition between nqp (red) and lcp (blue) phases. The interfacial interactions transmitted by the fluctuation of the Fe−N(≡C−Pd) coordination bonds, slippage of the layer structures, and π–π interactions between the prentrz ligands in different layers facilitate the water-adsorption induced nqp to lcp gate-opening transition.

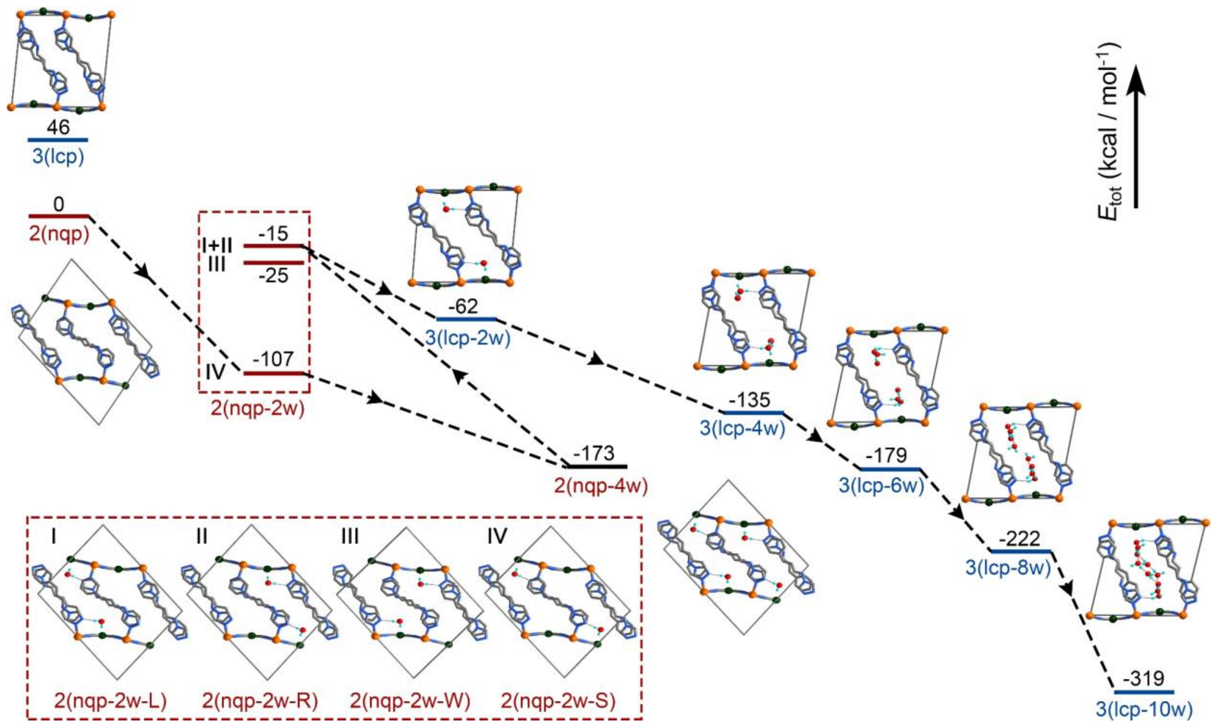

Supplementary Fig. 34 The energy diagram of water adsorption. The nqp (completed desolvated sample) to nqp-2w-S (state IV), and further to nqp-4w is energetically preferred water adsorption process, while the nqp-4w to lcp-4w experiences a simultaneous pore opening and closing process with an energy-uphill intermediated state of nqp-2w-L/R.

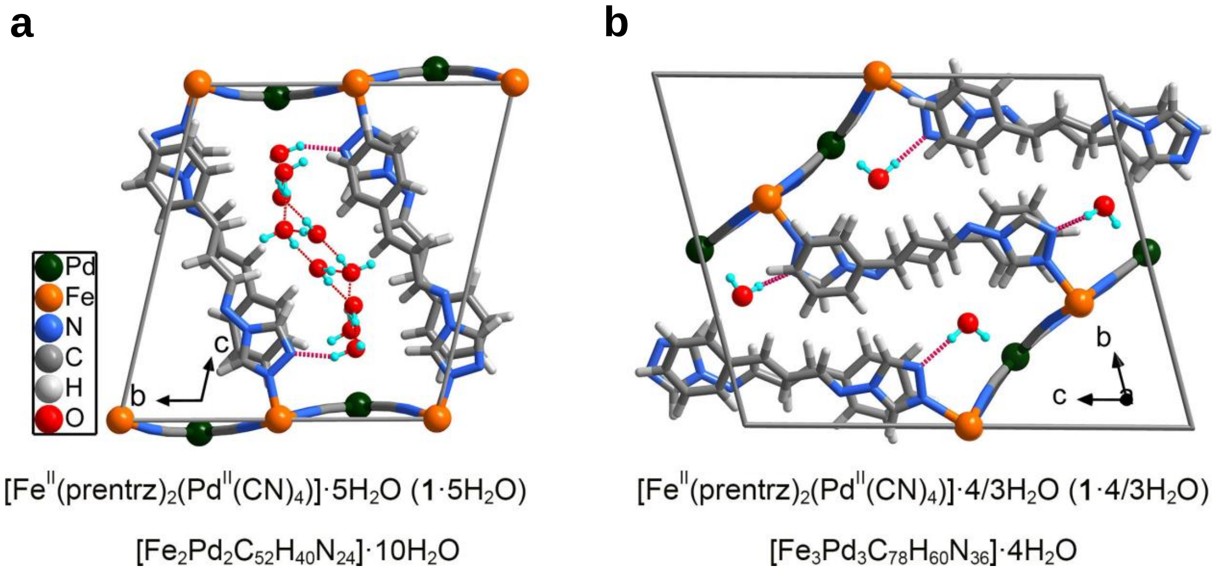

Supplementary Fig. 35 The unit cells of **1**·5H_2O and **1**·4/3H_2O used for calculation. (**a**) **1**·5H_2O. (**b**) **1**·4/3H_2O.

Supplementary Tables:

Supplementary Table 1 Crystal data and structural refinements for 1·9/2H_2O.

	**1**·9/2H_2O			
Temperature/K	293	250	150	85
Formula			$C_{52}H_{58}Fe_2N_{24}O_9Pd_2$	
Mr/g mol^{-1}			1487.72	
Crystal size/mm	0.05×0.08×0.03		0.1×0.15×0.03	
Wavelength/Å	0.77484		0.71073	
Space group	$P\bar{1}$	$P\bar{1}$	$P\bar{1}$	$P\bar{1}$
Crystal color	yellow	yellow	red	red
Crystal system	triclinic	triclinic	triclinic	triclinic
a/Å	7.4570(15)	7.4527(3)	7.2818(2)	7.1967(2)
b/Å	14.711(3)	14.7570(7)	14.5355(3)	14.2609(4)
c/Å	15.442(3)	15.4109(10)	15.3506(5)	15.2514(6)
α/°	104.49(3)	104.331(5)	104.332(2)	103.854(3)
β/°	99.92(3)	99.942(4)	100.229(2)	99.891(3)
γ/°	90.28(3)	90.138(4)	90.260(2)	90.132(2)
Volume/Å^3	1613.5(6)	1615.67(15)	1547.18(8)	1495.60(9)
Z	1	1	1	1
D_c / g cm^{-3}	1.531	1.529	1.597	1.652
μ / mm^{-1}	1.060	1.058	1.105	1.143
F(000)	754.0	754.0	754.0	754.0
θ range/°	1.432 to 25.0	3.89 to 25.03	3.50 to 30.00	3.53 to 29.92
Data/restraints/parameters	5185/18/418	5484/37/419	22636/6/419	21087/2/422
Goodness-of-fit on F^2	1.061	1.057	1.063	1.042
Reflections collected	10024	5684	22636	21087
R_1 [I ≥ 2σ(I)]	0.0631	0.0731	0.0570	0.0466
wR_2 [all data]	0.1826	0.1943	0.1897	0.1341
Largest diff. peak and hole/e.Å^{-3}	1.89/-1.41	4.74/-3.20	2.25/-1.30	1.04/-0.97
Completeness	91.5%	99.6%	99.6%	99.7%

Supplementary Table 2 Crystal data and structural refinements for 1·4/3H_2O.

	**1**·4/3H_2O	
Temperature/K	100	250
Formula	$C_{78}H_{68}Fe_3N_{36}O_4Pd_3$	
M_r/g mol^{-1}	2060.43	
Crystal size/mm	0.08×0.07×0.03	0.18×0.1×0.03
Wavelength/Å	0.77484	0.71073
Space group	$P\bar{1}$	$P\bar{1}$
Crystal color	red	yellow
Crystal system	triclinic	triclinic
a/Å	7.2100(14)	7.4258(5)
b/Å	16.097(3)	16.3023(11)
c/Å	19.051(4)	19.2325(12)
α/°	76.31(3)	76.768(6)
β/°	80.61(3)	80.084(6)
γ/°	79.15(3)	78.605(6)
Volume/Å^3	2093.5(8)	2202.0(3)
Z	1	1
D_c / g cm^{-3}	1.634	1.554
μ / mm^{-1}	1.210	1.150
F(000)	1036.0	1036.0
θ range/°	1.318 to 27.09	3.511 to 26.371
Data/restraints/parameters	7277/6/564	13208/18/500
Goodness-of-fit on F^2	1.129	1.032
Reflections collected	33449	13208
R_1 [$I \geq 2\sigma(I)$]	0.0881	0.0984
wR_2 [all data]	0.2671	0.2737
Largest diff. peak and hole/e.Å^{-3}	2.17/-2.33	4.94/-1.63
Completeness	89.0%	99.7%

Supplementary Table 3 Selected bond lengths and angles for 1·9/2H_2O at different temperatures.

	85 K	150 K	250 K	293 K
Fe1−N1	1.957(4)	1.957(5)	2.161(5)	2.154(5)
Fe1−N2	1.957(4)	1.952(4)	2.157(5)	2.159(5)
Fe1−N3	1.975(3)	1.982(4)	2.181(5)	2.164(5)
Fe2−N7	1.962(4)	2.145(4)	2.168(5)	2.152(5)
Fe2−N8	1.960(4)	2.160(5)	2.156(5)	2.171(5)
Fe2−N9	1.989(3)	2.175(4)	2.196(5)	2.185(5)
C1−N1−Fe1	178.5(4)	177.9(4)	178.9(6)	177.3(5)
C2−N2−Fe1	179.9(5)	179.4(5)	177.9(6)	178.7(5)
C3−N3−Fe1	129.2(3)	129.4(4)	129.2(3)	129.8(4)
C14−N7−Fe2	173.9(4)	163.4(4)	169.5(5)	163.8(5)
C15−N8−Fe2	171.3(4)	170.4(5)	164.9(5)	169.3(5)
C16−N9−Fe2	128.3(3)	125.6(4)	126.0(5)	126.4(4)

Supplementary Table 4 Selected bond lengths and angles for 1·4/3H_2O.

100 K		250 K	
Fe1−N1	1.940(5)	Fe1−N1	2.134(10)
Fe1−N2	1.941(6)	Fe1−N2	2.129(10)
Fe1−N7	1.965(6)	Fe1−N7	2.138(6)
Fe2−N3	2.059(5)	Fe2−N3	2.157(17)
Fe2−N4	2.039(7)	Fe2−N4	2.132(10)
Fe2−N5	2.052(8)	Fe2−N5	2.145(10)
Fe2−N6	2.061(7)	Fe2−N6	2.169(10)
Fe2−N11	2.096(8)	Fe2−N11	2.167(7)
Fe2−N15	2.062(6)	Fe2−N15	2.105(7)
C1−N1−Fe1	177.3(6)	C1−N1−Fe1	173.2(11)
C2−N2−Fe1	177.5(6)	C2−N2−Fe1	175.4(11)
C7−N7−Fe1	130.0(5)	C7−N7−Fe1	128.1(5)
C3−N3−Fe2	175.3(6)	C3−N3−Fe2	173.7(11)
C4−N4−Fe2	170.1(6)	C4−N4−Fe2	168.7(11)
C5−N5−Fe2	176.5(7)	C5−N5−Fe2	175.6(12)
C6−N6−Fe2	179.5(8)	C6−N6−Fe2	177.7(11)
C18−N11−Fe2	123.1(6)	C18−N11−Fe2	118.4(5)
C29−N15−Fe2	126.3(4)	C29−N15−Fe2	126.2(5)

Supplementary Table 5 Selected structural parameters for 1·9/2H_2O and 1·4/3H_2O at different temperatures.

**Compound**			**1**·9/2H_2O		**1**·4/3H_2O	
**Parameter**	85 K	150 K	250 K	293 K	100 K	250 K
<Fe1−N>[a]/Å	1.963(4)	1.964(4)	2.167(5)	2.159(5)	1.949(7)	2.134(9)
<Fe2−N>[a]/Å	1.970(4)	2.160(4)	2.173(5)	2.169(5)	2.062(3)	2.144(9)
∑Fe1[b]	11.24(14)	8.28(18)	14.0(2)	12.92(18)	6.4(15)	13.2(6)
∑Fe2[b]	9.24(15)	14.04(17)	7.2(2)	5.12(18)	19.4(11)	11.5(4)
d_{N-O}[c]	2.839(4)	2.847(4)	2.908(3)	2.896(4)	2.798(3)<br>2.940(4)	2.754(2)<br>3.018(5)
<Fe1−N−C>[d]	179.20(5)	178.60(5)	178.41(3)	178.01(5)	177.4(5)	174.25(5)
<Fe2−N−C>[d]	172.56(2)	166.93(5)	167.26(3)	166.62(4)	175.35(3)	173.90(3)
∑'[e]/°	4.72(6)	17.20(8)	7.46(5)	7.80(3)	5.043(12)	8.05(13)

[a] The average Fe–N bond lengths (Å);

[b] Octahedral distortion parameters (°);

[c] The distance of hydrogen bonds between uncoordinated nitrogen atom of 1,2,4-triazole in the prentrz ligand and oxygen atom of adjacent water;

[d] Average Fe–N–C angles within Hofmann layer;

[e] The parameter is defined as the average of the sum of $|90°-\theta|$ for Pd–Fe–Pd and Fe–Pd–Fe angles within the Hofmann layer (Three Pd–Fe1–Pd, three Pd–Fe2–Pd, two Fe1–Pd–Fe1, two Fe2–Pd–Fe2 and two Fe2–Pd–Fe1 angles of **1**·9/2H_2O; Three Pd–Fe1–Pd, five Pd–Fe2–Pd, two Fe1–Pd–Fe1, four Fe2–Pd–Fe2 and two Fe2–Pd–Fe1 angles of **1**·4/3H_2O).

Supplementary Table 6 Selected parameters for prentrz ligands of 1·9/2H_2O at different temperatures.

1-lcp	T/K	d/Å	θ1/°	θ2/°	θ3/°	θ4/°	θ5/°	θ6/°
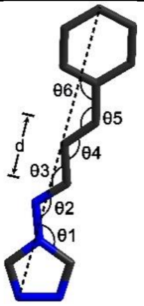	293	11.0(4)	133.8(3)	116.2(3)	119.8(4)	123.0(7)	127.3(1)	122.3(3)
	250	11.0(2)	133.0(3)	116.8(5)	118.8(8)	122.4(6)	126.9(4)	121.3(3)
	150	11.0(5)	132.4(2)	117.2(4)	118.9(3)	122.7(4)	126.7(3)	121.4(2)
**Conformer 1**	85	11.0(3)	132.3(5)	116.5(6)	118.6(2)	122.2(4)	125.9(4)	121.6(2)
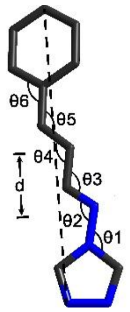	293	10.8(3)	120.8(1)	116.8(5)	119.9(4)	122.8(6)	127.0(1)	120.4(1)
	250	10.8(4)	120.8(2)	116.2(2)	119.1(4)	122.8(2)	126.9(6)	119.6(5)
	150	10.8(7)	121.1(4)	116.6(4)	118.4(5)	122.1(3)	126.2(3)	119.4(4)
**Conformer 2**	85	10.8(4)	120.4(6)	116.8(2)	117.9(2)	121.3(4)	126.4(7)	119.8(2)

Supplementary Table 7 Selected parameters for prentrz ligands of 1·4/3H_2O at 100 and 250 K.

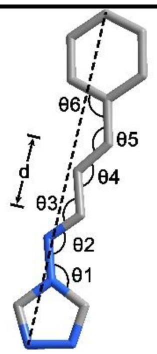

Conformer 1

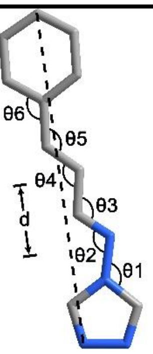

Conformer 2

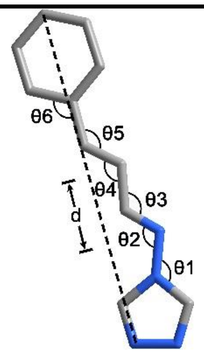

Conformer 2'

T/K	Parameter	d/Å	θ1/°	θ2/°	θ3/°	θ4/°	θ5/°	θ6/°
	**Conformer 1**	11.0(2)	133.3(5)	117.8(3)	118.2(3)	124.4(7)	127.2(2)	121.5(4)
**100**	**Conformer 2**	10.7(4)	121.0(3)	114.6(2)	119.0(4)	120.5(4)	126.8(5)	120.7(3)
	**Conformer 2'**	10.5(4)	122.7(6)	114.1(3)	124.0(5)	119.8(4)	130.6(7)	118.0(3)
	**Conformer 1**	11.0(3)	131.1(3)	119.3(4)	121.8(4)	125.8(4)	127.5(4)	120.6(5)
**250**	**Conformer 2**	10.7(3)	120.6(2)	116.8(3)	120.6(3)	121.7(4)	125.4(3)	118.3(3)
	**Conformer 2'**	10.6(3)	119.2(1)	112.6(5)	120.7(3)	117.6(2)	129.5(1)	118.2(2)

Supplementary Table 8 Comparison between experimental and calculated lattice parameters of 1·4/3H$_2$O and 1·9/2H$_2$O structures.

	**1**·4/3H$_2$O		**1**·9/2H$_2$O	
	crystal	PBE-D3	crystal	PBE-D3
a/Å	7.210	7.467	7.198	7.405
b/Å	16.097	15.761	14.262	13.940
c/Å	19.051	19.313	15.250	15.232
α/°	76.3	72.9	103.9	103.2
β/°	80.6	78.0	99.9	99.1
γ/°	79.2	75.6	90.1	89.6

Supplementary Table 9 The Gibbs free energy of each structure with ZPE correction and entropy correction at 300 K during the water-adsorption-induced porous transformation.

Crystal structure		Gibbs free energy (eV)
np	[$Fe_3Pd_3C_{78}H_{60}N_{36}$]	-1240.63
np-2w-strong	[$Fe_3Pd_3C_{78}H_{60}N_{36}$]·2$H_2O$	-1270.44
np-2w-weak	[$Fe_3Pd_3C_{78}H_{60}N_{36}$]·2$H_2O$	-1268.68
np-2w-left	[$Fe_3Pd_3C_{78}H_{60}N_{36}$]·2$H_2O$	-1268.46
np-2w-right	[$Fe_3Pd_3C_{78}H_{60}N_{36}$]·2$H_2O$	-1268.46
np-4w	[$Fe_3Pd_3C_{78}H_{60}N_{36}$]·4$H_2O$	-1299.39
lp	[$Fe_2Pd_2C_{52}H_{40}N_{24}$]	-826.42
lp-2w	[$Fe_2Pd_2C_{52}H_{40}N_{24}$]·2$H_2O$	-855.48
lp-4w	[$Fe_2Pd_2C_{52}H_{40}N_{24}$]·4$H_2O$	-884.04
lp-6w	[$Fe_2Pd_2C_{52}H_{40}N_{24}$]·6$H_2O$	-912.17
lp-8w	[$Fe_2Pd_2C_{52}H_{40}N_{24}$]·8$H_2O$	-940.30
lp-10w	[$Fe_2Pd_2C_{52}H_{40}N_{24}$]·10$H_2O$	-969.20
w	H_2O (adsorption state)	-13.75

Supplementary References

1. Scott, H. S., Ross, T. M., Moubaraki, B., Murray, K. S. & Neville, S. M. (2013). Spin crossover in polymeric materials using schiff base functionalized triazole ligands. Eur. J. Inorg. Chem. 803-812.
2. Hafner, J. (2008). Ab-initio simulations of materials using VASP: Density-functional theory and beyond. J. Comput. Chem. *29*, 2044−2078.
3. Perdew J. P., Burke K. & Ernzerhof, M. (1996). Generalized gradient approximation made simple. Phys. Rev. Lett. *77*, 3865-3868.
4. Grimme S., Antony J., Ehrlich S. & Krieg H. (2010). A consistent and accurate ab initio parametrization of density functional dispersion correction (DFT-D) for the 94 elements H-Pu. J. Chem. Phys. *132*, 154104.
5. Grimme S., Ehrlich S. & Goerigk, L. (2011). Effect of the damping function in dispersion corrected density functional theory. J. Comput. Chem. *32*, 1456−1465.
6. Wang, L., Maxisch, T. & Ceder, G. (2006). Oxidation energies of transition metal oxides within the GGA+U framework. Phys. Rev. B *73*, 1−6.
7. Kundu, T., Wahiduzzaman, M., Shah, B. B., Maurin, G. & Zhao, D. (2019). Solvent-induced control over breathing behavior in flexible metal–organic frameworks for natural-gas delivery. Angew. Chem. Int. Ed. *58,* 8073-8077.
8. Wang, V., Xu, N., Liu, J. C., Tang, G. & Geng, W. T. (2019). VASPKIT: A user-friendly interface facilitating high-throughput computing and analysis using VASP code, arXiv:1908.08269.
